# Modulation of pulmonary immune function by inhaled cannabis products and consequences for lung disease

**DOI:** 10.1186/s12931-023-02399-1

**Published:** 2023-03-28

**Authors:** Matthew Preteroti, Emily T. Wilson, David H. Eidelman, Carolyn J. Baglole

**Affiliations:** 1 Translational Research in Respiratory Diseases Program, Research Institute of the McGill University Health Centre, 1001 Decarie Blvd., Montreal, QC H4A 3J1 Canada; 2 Department of Pathology, McGill University, Montreal, QC Canada; 3 Department of Medicine, McGill University, Montreal, QC Canada; 4 Department of Pharmacology and Therapeutics, McGill University, Montreal, QC Canada

**Keywords:** Cannabis, COVID-19, Macrophages, Respiratory system, Endocannabinoids, Vape, Infection, COPD

## Abstract

The lungs, in addition to participating in gas exchange, represent the first line of defense against inhaled pathogens and respiratory toxicants. Cells lining the airways and alveoli include epithelial cells and alveolar macrophages, the latter being resident innate immune cells important in surfactant recycling, protection against bacterial invasion and modulation of lung immune homeostasis. Environmental exposure to toxicants found in cigarette smoke, air pollution and cannabis can alter the number and function of immune cells in the lungs. Cannabis (marijuana) is a plant-derived product that is typically inhaled in the form of smoke from a joint. However, alternative delivery methods such as vaping, which heats the plant without combustion, are becoming more common. Cannabis use has increased in recent years, coinciding with more countries legalizing cannabis for both recreational and medicinal purposes. Cannabis may have numerous health benefits owing to the presence of cannabinoids that dampen immune function and therefore tame inflammation that is associated with chronic diseases such as arthritis. The health effects that could come with cannabis use remain poorly understood, particularly inhaled cannabis products that may directly impact the pulmonary immune system. Herein, we first describe the bioactive phytochemicals present in cannabis, with an emphasis on cannabinoids and their ability to interact with the endocannabinoid system. We also review the current state-of-knowledge as to how inhaled cannabis/cannabinoids can shape immune response in the lungs and discuss the potential consequences of altered pulmonary immunity. Overall, more research is needed to understand how cannabis inhalation shapes the pulmonary immune response to balance physiological and beneficial responses with potential deleterious consequences on the lungs.

## Introduction

Cannabis, commonly referred to as marijuana, is a flowering plant belonging to the family of *Cannabaceae*. Cannabis constitutes a single diverse species called *C. sativa* L. with *C. sativa*, *C. indica*, and *C. ruderalis* being considered varieties [1]. *C. sativa* L. can be found in a variety of different habitats and altitudes, ranging from sea level to the foothills of the Himalayas [2]. Cannabis can be grown both indoors and outdoors and is dioecious (i.e., separate male and female plants). Cannabis is the third most prevalent psychoactive substance consumed after alcohol and tobacco [3–6]. Cannabis is also the most commonly-used illicit substance, with approximately 150 million users worldwide [7]. The psychoactive abilities of cannabis are due to the presence of the cannabinoid Δ^9^-tetrahydrocannabinol (Δ^9^-THC), but there are more than 100 other cannabinoids that could have alternative pharmacological properties. The most popular way to consume cannabis is smoking the dried plant in the form of a joint or using a water pipe. Cannabis smoke contains products of combustion, many of which are respiratory toxicants that could damage the lungs. Other methods of inhalation include vaporization which heats the dried plant material to generate a vapor. Heating without burning also releases cannabinoids but does not produce compounds caused by combustion. In the past few years, there has been increased interest in employing cannabis-based products as therapeutics, simultaneously with an increase in the flexibility in laws and regulations regarding the personal use of cannabis [8–10]. Due to legal restrictions however, over the last century, there are significant gaps in our understanding of how cannabis functions in the body, including how various inhaled products affect the pulmonary immune system. In this article, we provide an overview of the cannabis plant and its chemical components, the biology of the endocannabinoid system (ECS) and summarize current findings about the modulation of the pulmonary immune response from inhalation of cannabis products, including smoke and vapor. We further highlight the key areas where additional research is needed on this enigmatic plant and the consequences of its use.

## Historical advents of cannabis use

**Fig. 1:**
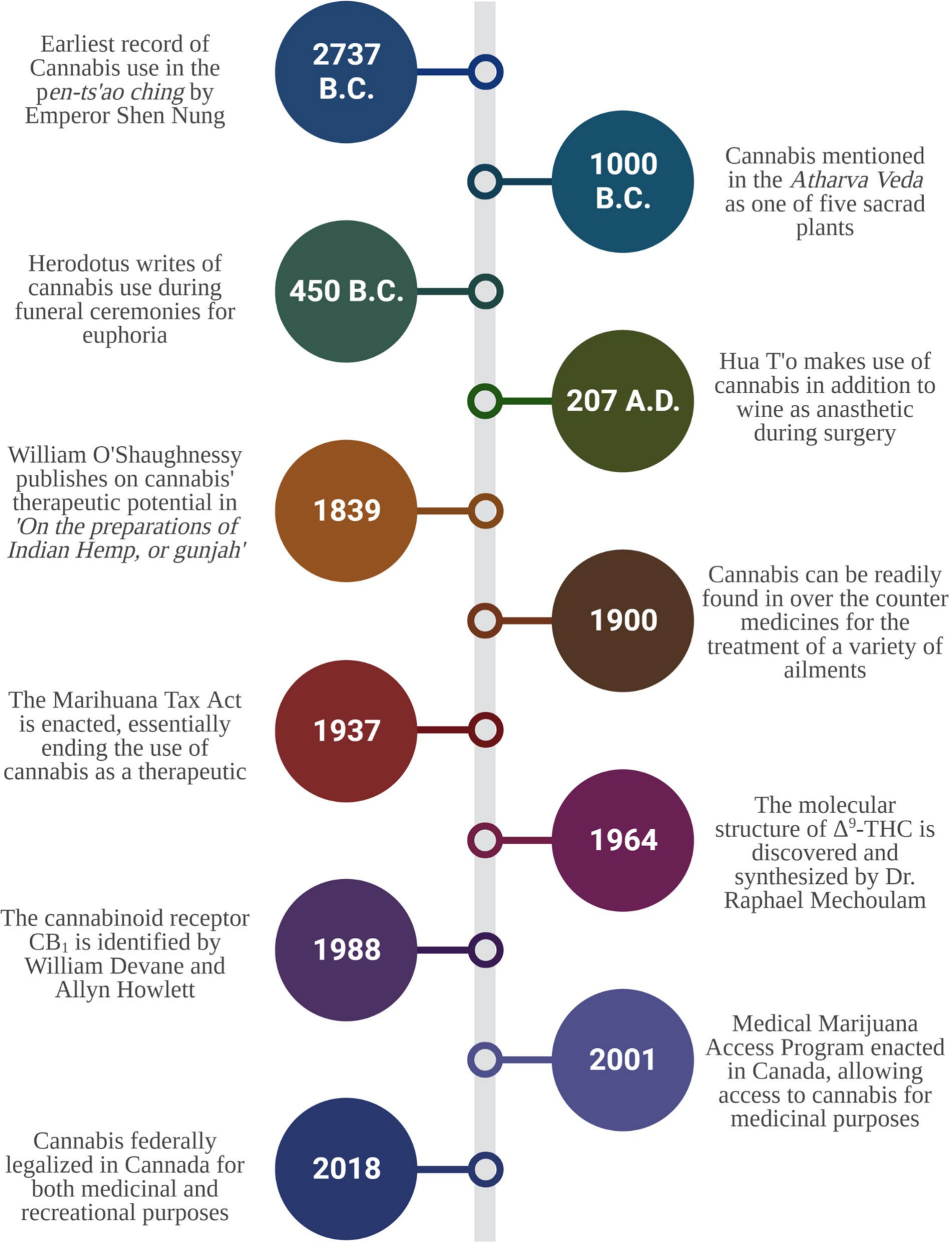
Timeline of cultural and medical milestones in cannabis. Summary of events beginning with the first recorded use of cannabis in 2737 B.C. up until the federal legalization of cannabis in Canada for both medicinal and recreational use

The cultivation of *Cannabis sativa* L. can be traced back at least 12,000 years, placing *C. sativa* L. among humanity’s oldest cultivated crops [11]. The earliest cultural evidence of cannabis use dates from 4800 BCE and involves the Yangshao, a Neolithic culture in China that appeared along the Yellow River Valley [11]. Cannabis stem-derived fibers were used to manufacture strings, ropes, textiles and paper, some of which have been discovered in the tomb of Emperor Wu (104-87 BCE) of the Han dynasty [11]. The first recorded use of cannabis as a medicinal drug was recorded in 2737 B.C. by the Chinese emperor Shen Nung in the world’s oldest pharmacopoeia, the *pen-ts’ao ching* [12], who documented its effectiveness in the treatment of pain associated with rheumatism, intestinal disorders, gout and malaria, among others (Fig. 1). Other ancient Chinese texts describe cannabis as a hallucinogen in the context of shamanism, which was widespread in regions of Central and Western Asia, as well as India, leading to an increase in cannabis use in those regions [13]. The medical and religious uses of cannabis in India can be dated to around 1000 BCE, with the plant having many applications including as an analgesic, anti-inflammatory, antibiotic and diuretic [14]. Other regions and cultures that have documented evidence of cannabis use prior to the Common Era include Tibet, where botany played a key component in their pharmacopeia. Cannabis, which was considered sacred, was very plentiful in that region and was commonly used in meditation [12]. There is also historical and archaeological evidence of cannabis use prior to the Common Era in Europe. Evidence suggests that the plant accompanied Scythian invaders that had originated in Central Asia and settled near the Mediterranean [15]. According to Herodotus, Scythians in the year 450 B.C. inhaled the vapors produced from burning cannabis seeds during a funeral ceremony for ritualistic and euphoric purposes. Consistent with this, archaeologists have discovered charred cannabis seeds in Scythian tombs [15]. Similarly, archaeologists unearthed thirteen female cannabis plants from an ancient tomb in northern China that were found lying diagonally across a man believed to be a shaman [16].

At the dawn of the Common Era, there are references to the use of cannabis seed-oil as a means to treat earaches and to deter insects [14, 15]. The use of cannabis in Africa has been documented since the fifteenth century and is believed to have originated from Arab traders that had a connection with India [17]. Uses of cannabis in Africa tended to differ from those of other cultures and regions, where its uses include snakebite, childbirth, malaria, asthma, and dysentery. In the same period, the medical use of cannabis remained very popular in India and had spread to regions in the Middle East and Africa. Cannabis gradually spread westward and was brought to Europe in 1150 initially to Spain and eventually to Italy [15]. In sixteenth century Europe, the use of cannabis was restricted to its cultivation for fibers, with very few references to the medicinal properties of the plant. The cultivation of cannabis in these regions has been documented in texts starting in the eighteenth century, with references to the distinctions between male and female cannabis plants [15].

European interest in the potential medical properties of cannabis was only kindled in the nineteenth century. In 1839, William O’Shaughnessy (Fig. 1), an Irish physician who tested its effectiveness in several pathologies, published on the effects of cannabis in animals and humans [18]. O’Shaughnessy noted that cannabis was capable of pain relief in patients, although it was not an effective treatment for cholera or rheumatism. In conditions characterized by muscle spasms such as tetanus and rabies, cannabis was able to ease spasticity [18]. Ultimately, the work by O’Shaughnessy [18–20] lead to wider interest in the medicinal properties of cannabis in the Western world. The first cannabis monograph was introduced into the 3rd edition of the American Herbal Pharmacopoeia in 1851, classifying cannabis as a botanical medicine. This increased interest led to 100 scientific articles being published in the latter half of the nineteenth century on the therapeutic effects of cannabis [21], including a report on the isolation of the first cannabinoid (cannabinol [CBN]) in 1899 [22]. During late nineteenth and early twentieth century, various laboratories were selling cannabis extracts. However, the medical use of cannabis tapered off in the early decades of the twentieth century due in part to the Marihuana Tax Act of 1937 (Fig. 1), and cannabis was removed from the American Herbal Pharmacopoeia in 1942. Today, the production and sale of cannabis has been legalized in many countries such as Australia, Canada, Chile, Colombia, Germany, Jamaica, the Netherlands, and Peru [8–10]. This has led to a resurgence of cannabis research and a better understanding of *C. Sativa* L. chemistry and its associated impact on human physiology.

## Chemical overview of *C. sativa* L.

With the increased legalization and social acceptance, cannabis has become a promising plant for medicinal use. However, regulations for cannabis remain stringent, largely because of the presence of Δ^9^-THC, the cannabinoid that causes psychoactive effects associated with its use. Cannabis is a chemically complex plant, and more than 500 compounds have been isolated from *C. sativa* L. [23, 24]. The general classes of these compounds—collectively referred to as secondary metabolites—include flavonoids, terpenes, as well as cannabinoids. Flavonoids are naturally occurring polyphenolic compounds that play multifunctional roles in the defense mechanisms of plants. Cannabis contains approximately 20 different flavonoids including cannflavin (A, B and C), vitexin, isovitexin and apigenin. Terpenes are aromatic organic hydrocarbons produced by a variety of plants and some insects. Terpenes are the primary constituents of essential oils and are responsible for determining how plants/fruits smell and protect plants by repelling insects and herbivores. Terpenes are used commonly as food additives and in cosmetic products such as soaps and perfumes [25]. Cannabis contains over 200 terpenes, largely monoterpenes and sesquiterpenes such as limonene (common to lemon and other citrus), α-pinene (common to rosemary and pine trees), linalool (common to lavender), β-Caryophyllene (found in black pepper) and α-bisabolol (found in chamomile).

**Fig. 2:**
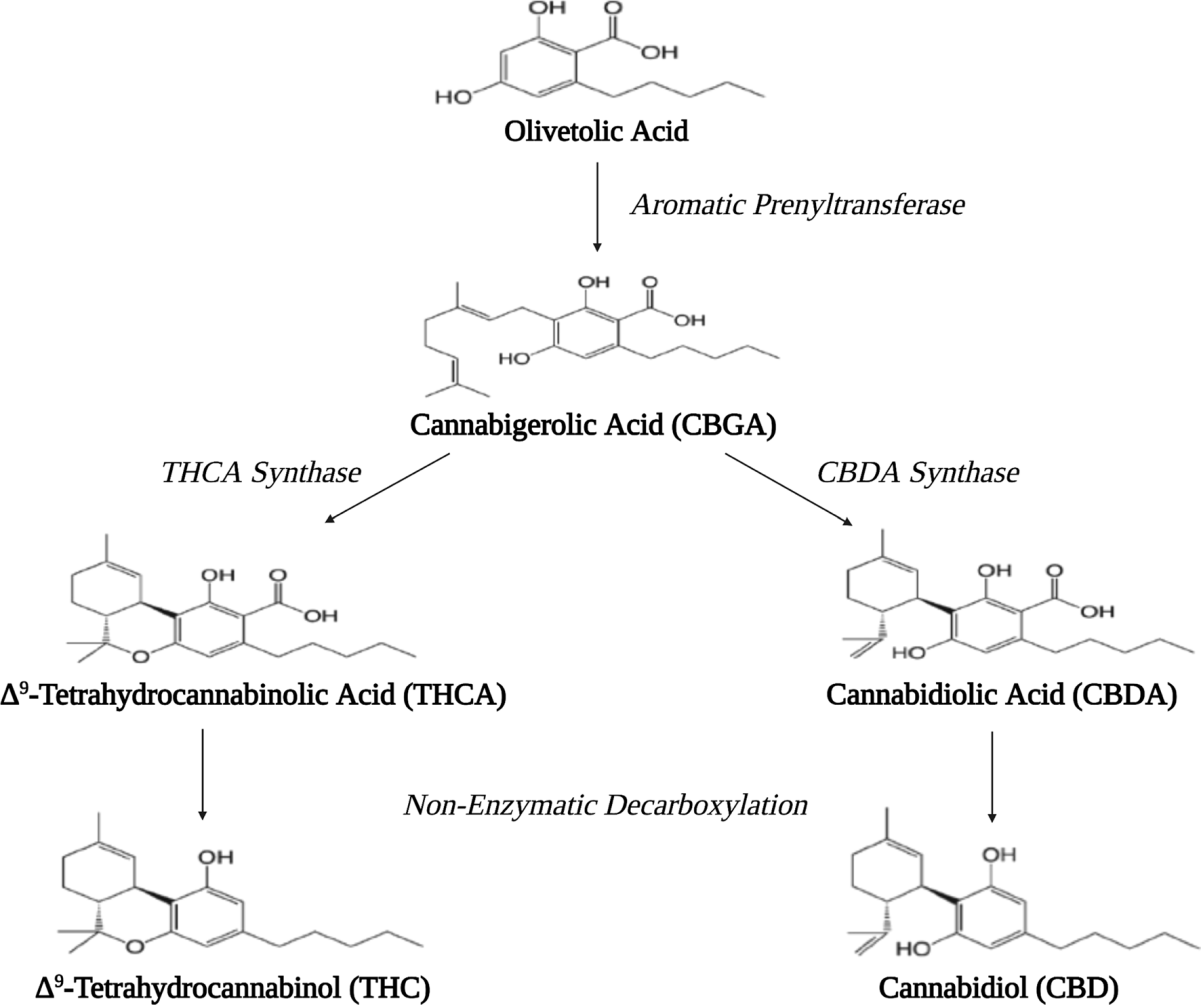
Biosynthesis of cannabinoids and structural differences between Δ^9^-THC and CBD. Conversion of olivetolic acid to cannabigerolic acid (CBGA) occurs through the use of aromatic prenyltransferase. CBGA acts as the point of differentiation from which cannabinoid-specific FAD-oxidases (THCA synthase & CBDA synthase) convert CBGA to precursor cannabinoid acids. Subsequent decarboxylation of cannabinoid acids results in active cannabinoids. Δ^9^-THC forms a cyclic ring whereas CBD has a hydroxy group resulting three-dimensional structural differences

Cannabinoids are terpenophenolic compounds with a ring structure derived from a C_10_ monoterpene subunit. The production of cannabinoids mainly occurs in the secretory head cells of the glandular trichomes [26] that are particulary concentrated in the bracts and flowers of the female inflorescence [27]. There are over 120 cannabinoids which are classified into 11 general types based upon their structure: Δ^9^-THC, Δ^8^-THC, cannabigerol (CBG), cannabichromene (CBC), cannabidiol (CBD), cannabinodiol (CBND), cannabielsoin (CBE), cannabicyclol (CBL), cannabinol (CBN), cannabitriol (CBT) and miscellaneous types [23]. The biosynthesis of cannabinoids follows the plastidial methylerythritol phosphate (MEP) pathway that has been described elsewhere and is summarized in Fig. 2 [28, 29]. Cannabis is used primarily because of its cannabinoids, and cannabis varieties can be differentiated based on their cannabinoid profile as being primarily Δ^9^-THC dominant or CBD dominant [30]. Δ^9^-THC was isolated in 1964 [31], a period that saw increased cannabis consumption throughout the western world. In 1967, the percentage of young adults who had used cannabis more than once was 5%; by 1971 usage increased to 44%, and by 1980 was 68% [32]. Today, cannabis is still the most commonly-used illicit substance, with approximately 150 million users worldwide [7].

## Cannabis consumption

There are a variety of ways in which people use cannabis, including oral (e.g., edibles, oils) and inhaled (vapor, aerosol, and smoke). However, the most common method of cannabis consumption (74% of users [33]) is inhalation of the smoke produced from combustion of the dried cannabis flower from a joint or water pipe [34]. Cannabis is the second most-smoked product after tobacco [35], although alternative methods of cannabis use are becoming popular, including vaporization, a technique that heats the dried plant without igniting it; both heating and burning release cannabinoids.

### Pharmacokinetics of inhaled cannabis

The bioavailability of cannabinoids differs depending on the route of consumption, with marked differences in bioavailability between oral and inhaled cannabis due to its lipophilic characteristics, poor aqueous solubility, and significant first-pass metabolism [36, 37]. Only 10–20% of Δ^9^-THC reaches the systemic circulation when taken orally [38]. Δ^9^-THC is almost instantly absorbed into the bloodstream after inhalation whereas absorption after oral ingestion can take an hour or more. Cannabinoids do exhibit similar pharmacokinetics (PK) regardless of whether they are generated from cannabis vapor or smoke [39]. After inhalation, Δ^9^-THC and CBD peak plasma concentrations have a rapid onset (3–10 min) [37, 40]. Bioavailability of inhaled Δ^9^-THC and CBD has high inter-subject variability due to variations in parameters such as puff duration, inhalation volume, inhalation device, and site of particle deposition [40, 41]. Once absorbed, cannabinoids initially distribute to highly vascular organs such as the lungs, heart, brain, and liver [42]. Next, cannabinoids distribute to the less vascularized tissue and finally, accumulate in adipose tissue due to their lipophilicity [40]. Subsequent release from adipose tissue can result in maintained cannabinoid activity for weeks after administration. Δ^9^-THC and CBD both have high volumes of distribution (10 L/kg and 3.4 L/kg, respectively) [40]. Metabolism of Δ^9^-THC and CBD occurs primarily in the liver via phase-I and phase-II enzymes [43]. Δ^9^-THC is metabolized primarily by CYP2C9 and CYP2C19 while CBD is metabolized by CYP2C19 and CYP3A4 [43]. Δ^9^-THC and CBD are eliminated through feces and urine. Both Δ^9^-THC and CBD have two compartment half-lives. Δ^9^-THC has an initial half-life of 4 h and a terminal half-life of 25–36 h [40]. CBD has an initial half-life of 1–2 h and a terminal half-life of 18–32 h [44].

## Biological activity of the endocannabinoid system

Once in the body, Δ^9^-THC and CBD exert their effects via the endocannabinoid system (ECS).The identification of the specific binding sites for Δ^9^-THC in the brain [45], complemented by the cloning of the cannabinoid receptor 1 (CB1), that led to the development of the concept of a ‘cannabinoid receptor system’ given the binding of Δ^9^-THC to CB1 as a partial agonist [46]. This was reinforced shortly afterward with the identification of the cannabinoid receptor 2 (CB2) [47], which in turn led to the discovery of the ECS [48]. The ECS is a widespread neuromodulator system that plays an important role in central nervous system (CNS) development and synaptic plasticity [49] as well as the regulation of sleep, mood, memory, appetite, reproduction, and pain sensation. The ECS comprises the cannabinoid receptors (CBRs), their endogenous ligands (endocannabinoids) and the enzymes/proteins involved their biosynthesis, degradation, and re-uptake.

### Endocannabinoids

Endocannabinoids are a family of bioactive lipids derived from arachidonic acid metabolism that activate CBRs. These endogenous cannabinoids include anandamide (AEA or *N*-arachidonoylethanolamide) and 2-arachidonoylglycerol (2-AG) [50–53]. In contrast to neurotransmitters, endocannabinoids are not stored in vesicles or cells but are synthesized on demand from lipid precursors in response to increases in intracellular calcium [54]. In the CNS, endocannabinoids mediate retrograde signaling that begins with the synthesis of 2-AG or AEA in the postsynaptic membrane. 2-AG or AEA is then liberated into the synaptic cleft and interact with CB1 receptors in the presynaptic membrane. Activated CB1 receptors block neurotransmitter release by inhibiting voltage-gated Ca^2+^ channels, decreasing presynaptic Ca^2+^ influx or through the adenylyl cyclase-mediated cAMP/protein kinase A (PKA) pathway [55, 56]. Finally, their actions are terminated by the degradation of 2-AG by monoacylglycerol lipase (MAGL) and AEA by fatty acid amide hydrolase (FAAH) [57].

### Cannabinoid receptors

2-AG is a high-efficacy agonist of the CB1 and CB2 with *K*_*i*_ values of 472 nM and 1400 nM, whereas AEA is a very low-efficacy agonist for CB1 and a low-efficacy agonist for CB2 receptors with *K*_*i*_ values of 5810 nM and 1930 nM, respectively [58, 59]. The exogenous cannabinoids Δ^9^-THC and CBD also interact with CBRs. Structurally, Δ^9^-THC and CBD share exactly the same molecular formula (C_21_H_30_O_2_), but differ in that Δ^9^-THC forms a cyclic ring whereas CBD forms a hydroxy group [60]. While Δ^9^-THC exists in a planar conformation, CBD adopts a conformation in which the two rings are at a right angle to each other. This molecular difference influences interactions with CBRs, such that CBD is unable to bind to CB1. Δ^9^-THC produces hypoactivity, hypothermia as well as spatial and verbal memory impairment via CB1 [61]. Conversely, CBD is non-psychoactive and does not regulate locomotor activity, body temperature or memory. Furthermore, activation of CB2 is devoid of any psychotropic effects [62].

CB1 and CB2 share 44% amino acid homology and are G protein-coupled receptors (GPCRs). Agonist binding results in a conformational change that causes the G-inhibitory (G_i_) alpha subunit to dissociate from the G beta-gamma (G_βγ_) dimer and the receptor. Upon release, the G_βγ_ subunit inhibits voltage-dependent calcium channels and activates inward rectifying potassium channels. Ultimately, activation of CB1 and CB2 results in the stimulation of mitogen-activated protein kinase (MAPK) activity and the inhibition of cyclic AMP (cAMP) production. CB1 is expressed throughout the CNS with expression being densest in areas of cognition and short-term memory as well as those regions associated with motor function and movement. CB1 receptors are expressed to a lesser degree in peripheral tissues but are present in the liver, thyroid, uterus, and bones [63]. The reported acute effects of cannabis on bronchodilation are attributed to the activity of Δ^9^-THC on the CB1 receptors of the axon terminals of postganglionic vagal nerves in the airway [64]. Conversely, the expression of CB2 within the CNS is minimal but has been detected under certain pathological conditions including nerve injury and inflammation. The expression of CB2 is present in organ systems such as the gastrointestinal system as well as immune tissue. Both CB1 and CB2 are expressed within the respiratory system [65] although comprehensive evaluation is lacking. CB2 expression may play a protective role the lungs. Preclinical studies show that CB2-deficient mice are susceptible to acute lung injury [66, 67] and that CB2 activation prevents airway epithelial permeability in vitro [68].

### Other GPCRs

The interaction of cannabinoids with endogenous receptors is not limited to CB1 and CB2 but includes other (orphan) GPCRs, including GPR5, GPR18, GPR55, GPR92 and GPR119 [69]. The pharmacology of these orphan receptors displays significant overlap with CB1 and CB2, particularly for GPR18 and GPR55, although our understanding of these receptors within the respiratory system remains limited. Cell-based studies indicate that GPR55 is activated by Δ^9^-THC, CBD, certain synthetic cannabinoids, and the endocannabinoids AEA and 2-AG [69]. GPR55 differs from CB_1_ and CB_2_ in that it is coupled to the G_12/13_α subunit rather than the G_iα_ subunit and increases levels of intracellular calcium upon activation [70]. Compared to CB_1_ and CB_2_, GPR55 has limited sequence homology of 14% and 15%, respectively [71]. However, cannabinoids such as Δ^9^-THC have weak GPR55 activity while CBD may be an antagonist of the receptor. GPR55 is highly expressed in the adrenals, the small intestine, and the CNS [70]. GPR18 is expressed within immune tissues such as the spleen, thymus, and lymph nodes [72]. Δ^9^-THC, CBD and anandamide act as partial agonists of GPR18 [73]. Of the previously mentioned GPCRs, GPR119 has the most limited homology with CB_1_ and CB_2_ receptors; however endogenous ligands that have demonstrated activity at these receptors also have activity for GPR119 [74]. GPR119 is expressed mainly in the brain, the pancreas and the gastrointestinal tract, where it is implicated in metabolism and glucose tolerance by acting on pancreatic beta-cells and intestinal endocrine cells [75].

### Other receptors

Cannabinoids can also interact with non-GPCRs, including the adenosine receptors, the vanilloid receptor 1 (TRPV1) and peroxisome proliferator activated receptors (PPARs). There are four known adenosine receptors—A1, A2A, A2B, and A3 [76]. Their primary ligand is the purine nucleoside adenosine, which functions in mitigating excessive cellular damage and inflammation during periods of acute stress [77]. Cannabinoids, including CBD have immunosuppressive effects through an inhibition of adenosine uptake, thereby promoting enhanced adenosine signaling [78]. The protective effects of adenosine are mediated by A2A receptors that can be found on virtually all immune cells [77]. Their activation leads to inhibition of T cell differentiation, downregulation of neutrophil superoxide production, as well as an inhibition of pro-inflammatory cytokine production [76].

TRPV1 is a homotetrameric membrane protein belonging to the transient receptor potential channel (TRP) family of which there are six known channels. This receptor is found predominantly within afferent sensory neurons and is involved in processes including body temperature, nociception, and detection of noxious environmental stimuli [79]. TRPV1 may be activated by several exogenous and endogenous stimuli. The best characterized activators include temperatures greater than 43 °C, low pH, capsaicin, and allyl isothiocyanate [80]. TRPV1 activation promotes the influx of calcium as a second messenger for the induction of pro-inflammatory cytokines and chemokines. Cannabinoids such as CBD can bind TRPV1 [80]. It has been proposed that the interaction between cannabinoids and TRPV1 leads to the desensitization of the receptor which subsequently contributes to an analgesic phenotype [80]. As such, the role of TRPV1 receptors in mediating the pharmacological effects of cannabinoids is of particular interest.

Finally, reporter gene assays have indicated that CBD and other cannabinoids activate nuclear PPARs (α, β and γ). PPARs are ligand-activated receptors that play diverse biological roles in energy homeostasis and fatty acid metabolism. PPARs can be found throughout the body, including in immune and structural cells. Although several studies indicate that PPARs mediate the anti-inflammatory effects of cannabinoids [81, 82], the mechanism is not definitively established.

## Cannabinoid modulation of immune function

Given their immune modulatory potential, the therapeutic potential of cannabinoids, particularly Δ^9^-THC and CBD, is of considerable interest. Cannabinoids reduce inflammation in a number of preclinical models of disease, including arthritis [83], multiple sclerosis (MS) [84, 85], inflammatory bowel disease (IBD) [86–89] and type 1 diabetes (T1D) [90–93]. Many of these effects are proposed to occur through CB2. Moreover, CB2 is expressed by various immune cells including B cells, macrophages, monocytes, natural killer (NK) cells, and T cells, suggesting that cannabinoids affect both the innate and adaptive immune systems. The effects of cannabis and select cannabinoids on immunological function has largely been assessed utilizing in vitro models; little is known about how inhaled products impact lung immunity specifically. A summary is provided below.

### Innate immunity

The innate immune system functions in collaboration with adaptive immunity in order to mount a full immune response to a pathogen or noxious substance. In the lungs, the innate immune system is comprised of physiological barriers (respiratory epithelium, mucus, surfactant) and immunological components, including resident/recruited immune cells. Below is an overview of the innate immune system and key features that are affected by cannabinoids.

### Natural killer (NK) cells

NK cells protect against infectious pathogens and limit the degree to which infection spreads via the termination of infected cells. NK cells are effector lymphocytes that possess qualities of innate and adaptive cells, including immunological memory, thus spanning both arms of immunity [94]. Few studies have investigated the effect of cannabinoids on NK cells. In response to Δ^9^-THC or CBD in vitro, there is an inhibition of the expression of inflammatory mediators including IL-8, MIP-1α, MIP-1β, RANTES, TNF-α, GM-CSF, and IFN-γ [95]. Δ^9^-THC can suppress NK cell function including cytolytic activity in rats, mice, as well as humans [96–98]. This effect is likely dependent on CB receptors, as Δ^9^-THC can inhibit NK cell cytolytic activity, which could be reversed by antagonists targeting either CB1 or CB2 [99].

### Neutrophils

Neutrophils are a short-lived cell with a half-life in the circulation of approximately 1.5–12.5 h in mice [100]. Neutrophils play important roles in early anti-microbial responses destruction through the release of proteins such as neutrophil elastase and matrix metalloproteinases (MMPs) as well as reactive oxygen species (ROS). Neutrophils are among the first cells recruited to the site of injury, including in the lungs in response to inhaled toxicants such as cigarette smoke. In addition, the lungs are a major neutrophil reservoir, and circulating neutrophils retained in the lung microvasculature are known as the lung-marginated neutrophil pool [101]. Despite being one of the first cells shown to express CB2, few studies have described how neutrophils respond to cannabinoids. It has, however, been shown that, cannabinoids induce the release of lysosomal enzymes from neutrophils in addition to modulating their response to chemokines [102]. Cannabinoids can also inhibit superoxide formation by neutrophils independent of CB1 or CB2 [103]. Thus, the current body of literature describing the interaction between cannabinoids and neutrophils is rather sparse and warrants further investigation.

### Mast cells

Mast cells are bone marrow-derived cells commonly found within connective and mucosal tissues. These cells play a predominant role in mediating inflammatory reactions, including allergic reactions such as asthma. Currently, the detection of CBRs on mast cells has been inconsistent. One study using human mast cells was unable to detect CB1 or CB2 but did demonstrate an ability to transport and release large quantities of AEA [104]. Conversely, two mast cell lines do express CB1 and CB2 at both the mRNA and protein level [105] and another study detected CB2 mRNA in rat peritoneal mast cells although administration of Δ^9^-THC dose-dependently released histamine irrespective of the CBRs [106]. Similarly, AEA, WIN 55212-2 or HU-210 can induce secretion of histamine in rat mast cells, an effect that was also independent of the CBRs [107]. Thus, more to research needs to be done with regards to the role of cannabinoids in mast cell immunology.

### Dendritic cells (DCs)

DCs are specialized antigen-presenting cells (APCs) that can initiate immune responses and contribute to the development of T cell-mediated immunity. Components of the ECS are present in DCs, including CB1 and CB2 in addition to anandamide, 2-AG and FAAH [108]. In vivo administration of Δ^9^-THC decreases the number of splenic DCs as well as reducing the expression of MHC II [109]. Δ^9^-THC and AEA induce apoptosis in murine bone marrow-derived DCs through an activation of caspases 2, 8, and 9. This effect was dependent on the engagement of the cannabinoids with both CB1 and CB2 [109]. Δ^9^-THC inhibits the differentiation of monocytes into antigen-presenting dendritic cells (DCs), preventing DCs from stimulating T cell proliferation or maturing into functional effector/memory T cells [110]. Collectively, these studies suggest that DCs may be important peripheral targets for cannabinoids.

### Macrophages

**Fig. 3:**
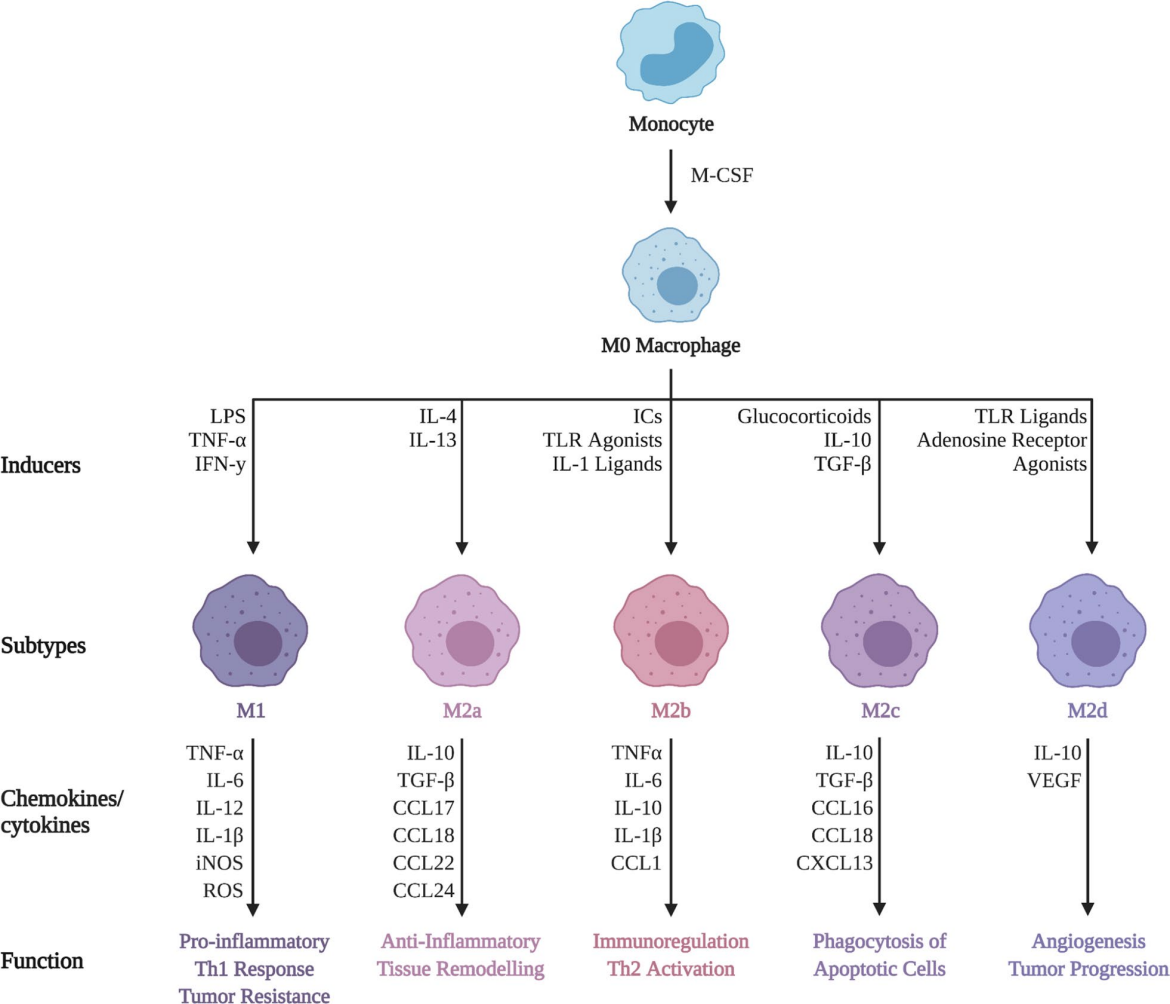
Differing biological and physiological features of macrophage subsets. Monocytes stimulated by macrophage-colony stimulating factor (M-CSF) differentiate into M0 macrophages. M0 macrophages subjected to certain stimuli promote a phenotype of either M1, M2a, M2b, M2c, or M2d. Each phenotype has characteristic cytokine/chemokine secretion profiles with respective cellular and molecular functions; adapted from [196]

Macrophages are a heterogeneous population that are positioned throughout the body to facilitate the ingestion of dead cells, debris, foreign material, and the orchestration of inflammatory processes [111]. Macrophages are also APCs that capture, endocytose and present self or foreign antigen on the cell surface to facilitate an adaptive immune response. Macrophages typically exist in two subsets: classically-activated macrophages (M1) or alternatively-activated macrophages (M2) [112]. M1 macrophages are pro-inflammatory and produce cytokines such as IL-1β, IL-6, IL-12, and TNF-α. M2 macrophages are anti-inflammatory and produce cytokines such as IL-10 and TGF-β to suppress inflammation, and contribute to tissue repair and remodeling [112]. M2 macrophages can be further subdivided into M2a, M2b, M2c, or M2d (Fig. 3). The M2a phenotype can be induced by IL-4 or IL-13 and produce high levels of CD206, decoy receptor IL-1 receptor II (IL-1RII) and IL-1 receptor antagonist (IL-1Ra) which function to promote tissue remodeling. The M2b phenotype can be induced by stimulation with immune complexes (ICs), toll-like receptor (TLR) agonists, or IL-1 receptor ligands. The M2b subset most accurately reflects the intermediate phase between M1 and M2 with the release of both pro- and anti-inflammatory cytokines including IL-10, IL-1β, IL-6 and TNF-α which promote a Th2 response as well as tissue remodeling. M2c macrophages can be polarized by glucocorticoids in addition to IL-10 and exhibit anti-inflammatory properties against cells undergoing apoptosis via the release of IL-10 and TGF-β. Finally, macrophages with a M2d phenotype are induced by TLR agonists through the adenosine receptor; this leads to a reduction in the secretion of pro-inflammatory cytokines and an increase in the secretion of anti-inflammatory cytokines.

In vitro studies support the notion that the function and polarization of macrophages is significantly altered by cannabinoids and/or manipulation of CBRs. In this regard, CB1 activation suppresses M2 macrophage polarization [113]. Furthermore, selective CB2 activation reduces M1 macrophages in favor of M2 polarization [114] and increases efferocytosis of apoptotic cells [115]. In peritoneal macrophages, Δ^9^-THC downregulates nitric oxide (NO) production as well as TNF-α maturation and secretion [116]; Δ^9^-THC can also impair the phagocytic activity of M2 macrophages [117]. Evidence for the influence of CBD on macrophage function is sparser. A study of the human monocytic cell line U-937 revealed differential effects of CBD on IL-8, macrophage chemoattractant protein (MCP)-1, and cellular ROS levels. Following induction by LPS, CBD attenuated IL-8 and MCP-1 production but at the basal level, CBD induced the production of IL-8, CXCL1, Serpin E1, IL-6, IFN-y, MCP-1, RANTES, and TNF-α, indicating that the effects of CBD may depend on the activation state [118] or anatomical location of the cells. This may be particularly true for pulmonary macrophages, given that they are the first to encounter inhaled cannabinoids and toxicants from combustion.

### Adaptive immunity

Adaptive immunity protects against specific infectious organisms and is carried out primarily by B- and T-lymphocytes that confer immunological memory. Δ^9^-THC and CBD affect both B-and T-cell function, including proliferation, survival, and antibody production [119–122].

### T cells

Δ^9^-THC inhibits proliferation of human lymphocytes in culture and leads to apoptosis of murine macrophages and T cells through the regulation of Bcl-2 and caspase activity [122]. The effects of Δ^9^-THC are significantly greater in naïve rather than activated lymphocytes, possibly the result of decreased CB2 expression in activated cells [123]. This was further investigated in a study that made use of the CB_2_ agonist JWH-015 [124] which not only inhibited proliferation, but also induced apoptosis in naïve- and activated thymocytes. CBD, at concentrations in the micromolar range, induces apoptosis in CD4^+^ and CD8^+^ T cells in a time- and dose-dependent manner [125]. Δ^9^-THC can also increase apoptosis in activated T cells while simultaneously increasing the number of T regulatory (T_regs_) cells. Similarly, Δ^9^-THC and CB_2_ agonists can suppress the differentiation of monocytes into antigen-presenting DCs which results in an inability of DCs to stimulate T cell proliferation or to promote their differentiation into functional effector/memory T cells [110]. This may explain how cannabinoids reduce features of diseases like MS in animal models. Administration of JWH-015 to mice reduced microglial activation, abrogated MHC II antigen expression, and decreased the number of CD4^+^ T cells infiltrating the spinal cord. Recovery of motor function and reduction in inflammation were also observed along with extensive remyelination following JWH-015 [84]. In patients with MS, the novel CB2-selective agonist COR167 reduced the proliferation of both peripheral blood mononuclear cells (PBMCs) and myelin basic protein-reactive T cells in a dose-dependent manner [126].

### B cells

B cells express high levels of CB2, and several studies have investigated the potential of cannabinoids to modulate B cells, the antibody-producing cells of the adaptive immune system. Several reports have shown that cannabinoids exert a variety of effects on B cells including altered proliferation as well as reduced antibody production [119]. In contrast, Δ^9^-THC and the synthetic cannabinoid analog WIN 55212-2 promote B cell proliferation [120]. Both Δ^9^-THC and CBD may protect against cell death independent of CB1 and CB2 [121]. Endocannabinoids, including 2-AG, may stimulate the migration in addition to inducing B cell differentiation [127]. It remains to be established whether these effects are the result of direct interaction with B cells, or an indirect effect mediated through T cells or the innate immune system.

## Pulmonary consequences of cannabis inhalation

**Fig. 4:**
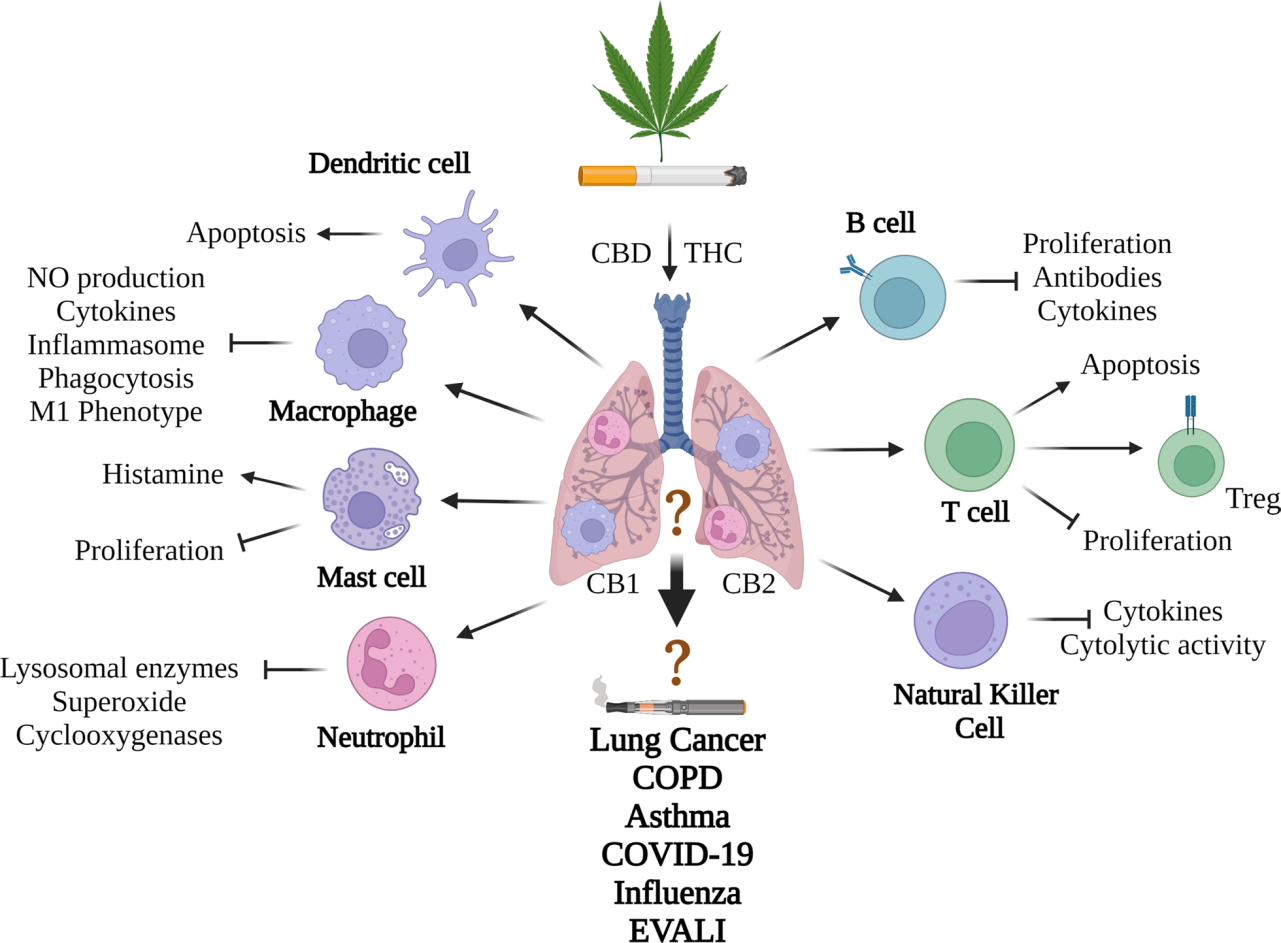
Summary of how cannabis products may impact the pulmonary immune system. Smoking cannabis increases inflammation in the lungs that is typified by increased neutrophils and macrophages. Mechanistically, the direct impact of the cannabinoids THC and CBD on various immune populations is shown although whether these are also impacted in the lungs from inhaled cannabis products is not known. Generally, cannabinoids are immunosuppressive, and prevent cytokine production, proliferation and cell-specific functions (e.g., phagocytosis, antibody production). In some cases, cellular differentiation to a more suppressive phenotype (e.g., Tregs) is observed. The functional consequence of these immunological changes is not known but is theorized to contribute to chronic lung disease development including lung cancer and COPD. The impact on respiratory infections is inconclusive. It is also not known the impact of vaporized cannabis products, including the inhalation of distillates using an e-cigarette. More research is needed to understand the evolving and complex interaction between inhaled cannabis products and the pulmonary immune system

In addition to the release of cannabinoids, smoking cannabis also generates a myriad of pyrogenic compounds, including carcinogens, mutagens, and teratogens, that have the potential to cause adverse health outcomes [128]. These compounds are similar to those found in cigarette smoke [128]. Cigarette smoke and cannabis smoke have 231 compounds in common, with 69 of these being toxic [128]. In contrast to cigarette smoke, the effects of cannabis smoke on the pulmonary system are much less well understood. A major challenge is that many cannabis smokers also use tobacco products [129]; almost 90% of individuals who smoke cannabis also smoke tobacco cigarettes [130]. Moreover, there are differences in how people inhale cannabis smoke compared to tobacco smokers. Cannabis smokers take larger puffs, inhale more deeply, and hold their breath four times longer, which leads to a different deposition of particles and increased tar deposition [131, 132]. Despite being in direct contact with inhaled compounds, the impact of cannabis smoke on pulmonary immunity remains poorly understood, with much of the information centered on assessment of immune cell recruitment. In the previous section, we highlighted how cannabis may impact immune function. Below, we provide the current-state-of knowledge on how the smoking of cannabis affects immunity in general and within the pulmonary system where data is available; a collective summary of these findings can be seen in Fig. 4.

### Cannabis smoke

#### Impact on the immune system

Given the similarities in combustion products between cannabis smoke and cigarette smoke, it has been theorized that there are similar inflammatory effects in the lungs from cannabis smoke. Evidence in support of this comes from studies in human cannabis smokers in whom the number of neutrophils and macrophages are increased when compared to non-smokers [133, 134]. The increase in macrophages is thought to be the result of tissue-infiltrating monocytes migrating in response to the inhaled smoke [135]. This may have important consequences, as different macrophage subtypes occur in the healthy lung. The lung has two macrophage populations: alveolar macrophages and interstitial macrophages (IMs), which differ based on origin and function. Alveolar macrophages are long-lived, embryonically-derived cells that self-renew to maintain their population [136]. In response to injury, bone marrow monocytes also migrate to the lungs and differentiate into macrophages to restore the alveolar macrophage pool. The main functions of alveolar macrophages are to recycle pulmonary surfactant, protect against infectious organisms and efferocytose apoptotic cells to prevent tissue damage. IMs on the other hand are thought to have a mixed origin, being initially derived from yolk sac precursors, and later replaced by circulating monocytes. There are three subsets of IMs (IM1, IM2 and IM3) and all are generally immunosuppressive/tolerogenic due to their constitutive production of IL-10 [137, 138]. Chronic cannabis smoke inhalation in animal models leads to recruitment of immune cells to the lungs, including macrophages [139]. Furthermore, cannabis smoke alters the percentage of macrophage subpopulations within the lungs by increasing both tissue-resident and monocyte-derived alveolar macrophages as well as the IM1 subpopulation in male mice [140]. Phenotypically, macrophages from cannabis smokers are significantly enlarged and contain large amounts of inclusion bodies and particulates consistent with tar [141]; these macrophages also contain Δ^9^-THC and Δ^9^-THC metabolites [134].

Like cigarette smoke, exposure to cannabis smoke results in functional impairment in macrophages. When challenged with a common respiratory pathogen such as *Staphylococcus aureus* (*S. aureus*), alveolar macrophages from cannabis smokers are deficient in both bacterial phagocytosis and killing [142]. The reduced phagocytotic ability of alveolar macrophages is marked by reduced oxygen consumption and superoxide formation. Similarly, alveolar macrophages from cannabis smokers exhibited reduced production of NOS, TNF-α, GM-CSF, and IL-6 when compared to non-smokers as well as tobacco smokers [142]. Incubation of these cells with GM-CSF or IFN-y restores NOS production, suggesting that cannabis exposure causes a decrease in cytokine priming that weakens host defense. As such, there is evidence to support an immune-suppressive effect of cannabis smoke that may impair anti-microbial defenses, similar in nature to the alteration of macrophage function to individual cannabinoids noted above.

#### Respiratory symptoms and chronic lung disease

Studies suggest that smoking cannabis is associated with worsening respiratory symptoms, including cough or sputum production, wheezing, and shortness of breath [143]. Other reported acute effects of smoking cannabis include bronchodilation whereas chronic use may lead to increased large airway resistance [64]. The implications of these findings in those with chronic lung disease is unclear. For example, in people with asthma, an obstructive lung disease that affects nearly 300 million people globally [144], the effect of cannabis on lung function is contradictory. Some studies indicate that cannabis smoking exerts bronchodilation [145] while others report that cannabis increases symptoms of asthma [146].

The effects of smoking cannabis on the development of other lung diseases largely attributed to cigarette smoke, including lung cancer and chronic obstructive pulmonary disease (COPD), is unclear and contradictory [147]. COPD is a prevalent disease characterized by progressive, irreversible airflow obstruction caused by inhalation of noxious particles; the main cause of COPD is cigarette smoke. Studies on COPD and cannabis smoke are conflicting in humans, with some suggesting a synergy with tobacco [148] and others showing no association [149]. Few studies have found changes in lung function (FEV_1_/FVC) or emphysema in cannabis users although symptoms of chronic bronchitis (cough, sputum and wheeze) are noted [150]. There remains a lack of convincing data on the chronic cannabis smoke inhalation and development of alveolar damage [151] and remains equivocal owing in part to varying definitions of “joint years”, variation in usage patterns between cannabis and tobacco smokers, changes in the size and strength of cannabis cigarettes (“joints”) over time and control for concomitant tobacco use. [130, 151, 152]. There also remain a limited number of preclinical studies on cannabis smoke inhalation, with existing data supporting a link between chronic cannabis smoke and an emphysema-like phenotype [139]. Mechanistically, the effects of cannabis smoke may be partially related to an effect on alveolar macrophages. Although cigarette smoke increases the number of alveolar macrophages, paradoxically cigarette smoke also reduces clearance of apoptotic lung epithelial cells and neutrophils by macrophages. This failure to resolve the inflammatory response may lead to emphysema due to secondary necrosis [153–155]. Cannabis smoke also functionally impairs the phagocytic capacity of alveolar macrophages [142]. Given the immunosuppressive features of cannabis smoke on lung macrophages, a similar response could occur, although more research is needed to understand the impact of cannabis smoke and cannabinoids on macrophage efferocytosis and whether such changes are beneficial or detrimental.

The link between lung cancer and cannabis smoke inhalation is equally unclear. Regular cannabis users show histopathologic precursors to malignancy development, findings that are consistent with non-human primate models [156]. However epidemiological, case–control, and cohort studies have shown no increased risk of cancer from  smoking cannabis [133, 157–159] and there is a lack of DNA adducts in animal models of chronic cannabis exposure [160]. Thus, current evidence linking cannabis smoke inhalation and chronic lung disease development remains limited [161, 162].

#### Respiratory infections

Respiratory infections cause significant morbidity and mortality worldwide, both in the form of primary infections (e.g., pneumonia) as well as exacerbations (worsening of symptoms) of chronic lung conditions such as COPD [163]. Cigarette smoke is well described to be a key risk factor for numerous infectious diseases, including latent and active tuberculous [164] as well as increased risk of severe influenza [165–168]. Evidence that cannabis smoke alters susceptibility to respiratory infection is lacking but its overall effect on immune function as noted above suggests the cannabis smoking may predispose susceptible individuals to pulmonary infections, including those that are immune compromised by human immunodeficiency virus (HIV) or cancer chemotherapy [133, 169].

The recent coronavirus disease-2019 (COVID-19) pandemic, caused by the severe acute respiratory syndrome-coronavirus-2 (SARS-CoV-2) virus, raised the question as to whether cannabis had an impact on outcomes. A number of factors have been shown to increase risk of severe illness after infection with the SARS-CoV-2 virus, including age and underlying chronic medical conditions including cancer, COPD, cardiovascular disease, and conditions causing an immunocompromised state [170, 171]. The presence of underlying medical conditions increases the fatality rate from 0.9 to 10.5% [172]. COVID-19 is associated with a myriad of symptoms ranging from asymptomatic to severe pneumonia and acute respiratory distress syndrome (ARDS) leading to death [173]. Evidence that cigarette smoke increases the risk of SARS-CoV-2 infection is inconsistent [174] although there is indication that chronic cigarette smoking increases the pulmonary expression of the angiotensin converting enzyme 2 (ACE2), the entry receptor for SARS-CoV-2 [175]. This effect may be reversed by smoking cessation [176]. The impact of cannabis smoke on SARS-CoV-2 infection and development of COVID-19 is not known, but experimental evidence suggests that CBD alone decreases ACE2 expression [177] and inhibits SARS-Cov-2 replication [178]; there is also evidence that CBD reduces COVID-19 related inflammation [179]. Studies utilizing over 800 *C. sativa* strains in 3D human models of COVID-19 target tissues (oral, airways, and intestinal) noted that high CBD/low THC extracts downregulate ACE2 gene and protein levels [171, 177, 180]. Finally, CBD can significantly inhibit SARS-Cov-2 replication in mouse models of infection, thereby reducing the viral load in the lungs and reducing signs of clinical disease [178]. While these experimental studies demonstrate that cannabinoids can affect lung damage and inflammation caused by infectious organisms, the route of administration is an important variable that may impact outcomes and does not often represent human consumption patterns where cannabis is most often inhaled. More research is needed to understand how cannabis smoke impacts lung injury caused by respiratory infections.

#### Acute lung injury (ALI)/acute respiratory distress syndrome (ARDS)

Both viral and bacterial agents can cause acute lung injury (ALI)/acute respiratory distress syndrome (ARDS). ALI occurs where there is acute inflammation and disruption of the vascular endothelium and the alveolar epithelium, leading to loss of alveolar-capillary membrane integrity, excessive neutrophil transmigration and the release of pro-inflammatory mediators, culminating in respiratory failure [181]. Cannabinoids may be efficacious in reducing inflammation in the context of ALI. For example, in a model of ALI, prophylactic treatment with CBD reduces inflammation as well as total lung resistance and elastance [182]. Additionally, in an endotoxin B-induced model of lung inflammation in mice, Δ^9^-THC given through intraperitoneal injection decreased mortality, vascular leakage, leukocyte infiltration, as well as the concentration of pro-inflammatory cytokines [183]. Similarly, Δ^9^-THC has a therapeutic effect in an LPS-induced model of ALI in mice whereby intranasal administration significantly reduced TNF-α levels as well as the number of infiltrating neutrophils [184]. Oral administration of CBD, however, enhances LPS-induced pulmonary inflammation [185]. Finally, administration of CBD intraperitoneally reduced proinflammatory cytokines in an animal model of ARDS induced by a viral mimetic [186], suggesting a potential benefit in the treatment of lung inflammation/injury from COVID-19.

### Vaporized cannabis

While most people consume cannabis via inhalation of cannabis smoke, vaporizing cannabis (heating the dry plant material) is increasingly popular [187, 188]. Pyrogenic compounds noted above are predicted to be absent or highly reduced in vaporized cannabis [128]. As such, vaporizing cannabis is predicted to be a safer alternative to cannabis smoking. The “Lower-Risk Cannabis Use Guidelines” from the Centre for Addiction and Mental Health (CAMH) recommend vaporizers as an alternative to smoking to avoid respiratory problems [189]. In a single existing study comparing smoking to the vaporization of cannabis, exhaled carbon monoxide (eCO) was measured as an indicator of inflammation [190]. Here, smoked cannabis significantly increased eCO while exposure to vaporized cannabis showed no significant increase in eCO [191]. In a small, non-randomized clinical trial, individuals who regularly smoked cannabis and reported respiratory symptoms were asked to switch to cannabis vapor for 1 month. At the completion of the trial, test subjects had a significant improvement in respiratory symptoms as well as forced vital capacity indicating the reduced risk of vaporized cannabis compared to the smoked product [192].

There are also reports of ALI associated with cannabinoid oil vaping with no clear mechanism of injury [193]. Here, oils containing THC or CBD are heated using an electronic vaporizer (e-cigarette), like for nicotine-containing products. The overall composition of the oils may play a direct role in this toxicity, as these case reports are similar to the e-cigarette product use associated lung injury (EVALI) outbreak that was accompanying by adverse respiratory symptoms including shortness of breath, chest pain, cough and in severe cases death. The majority of EVALI cases occurred in the United States, and was strongly linked to e-cigarettes containing Δ^9^-THC where vitamin E acetate (VEA) was used as a diluent in black-market products [194]. Subsequent experimental evidence supports a causative role for VEA in the development of EVALI-like symptoms in rodent models [195]. While these recent data show that there may be a benefit of vaporizing cannabis over combustion, almost nothing is known about the ability of vaporized cannabis to modulate immune function in the respiratory system and affect the downstream development of lung diseases.

## Conclusions and future perspectives

Despite its long history of human use, issues of legality have contributed to significant gaps in our understanding of the efficacy of cannabis in alleviating symptoms of disease and the physiochemical properties of the more 500 chemicals that cannabis produces. With increased legalization and wider social acceptance, cannabis has become a promising plant for medicinal use. Alongside this greater acceptance is an increasingly diverse portfolio of products, many of which are designed to be inhaled and thus encounter resident and recruited immune cells. Evidence from experimental in vitro models indicates that cannabinoids modulate facets of both innate and adaptive immunity, many of which are immunosuppressive, and this could be beneficial in certain scenarios but detrimental in others. Moreover, many of the cannabinoid-specific effects are overshadowed by the myriad of additional chemicals products during combustion of the cannabis plant. Understanding the immunological consequences of inhaled cannabis products is further complicated by the diversity of products, lack of standardized preclinical models that recapitulate human use patterns and the variation in THC levels that has occurred in cannabis over time. Future research focused on phytochemical interaction with the ECS, the safety and efficacy of new and emerging products, in conjunction with its spectrum of potential medical applications and impact on economic and legal policy, can shed much needed light on this enigmatic plant.

## Data Availability

Not applicable.

## Abbreviations

2-AG: 2-Arachidonylglycerol
A2A: Adenosine A2A receptor
ACE2: Angiotensin converting enzyme 2
AEA: Anandamide
ALI: Acute lung injury
APC: Antigen-presenting cell
ARDS: Acute respiratory distress syndrome
cAMP: Cyclic adenosine monophosphate
CB1: Cannabinoid receptor 1
CB2: Cannabinoid receptor 2
CBC: Cannabichromene
CBD: Cannabidiol
CBE: Cannabielsoin
CBG: Cannabigerol
CBGA: Cannabigerolic acid
CBL: Cannabicyclol
CBN: Cannabinol
CBND: Cannabinodiol
CBR: Cannabinoid receptor
CBT: Cannabitriol
CNS: Central nervous system
COPD: Chronic obstructive pulmonary disease
COVID-19: Coronavirus-induced disease-19
DC: Dendritic cell
eCO: Exhaled carbon monoxide
ECS: Endocannabinoid system
EVALI: The e-cigarette product use associated lung injury
FAAH: Fatty acid amide hydrolase
G_i_: G-inhibitory alpha subunit
GPCR: G-protein coupled receptor
G_βγ_: G beta-gamma dimer
IBD: Inflammatory bowel disease
IFN: Interferon
HIV: Human immunodeficiency virus
K_i_: Inhibitory constant
IM: Interstitial macrophage
LPS: Lipopolysaccharide
MEP: Methylerythritol phosphate
M1: Classically activated macrophages
M2: Alternatively activated macrophages
MAGL: Monoacylglycerol lipase
MAPK: Mitogen-activated protein kinase
MCP-1: Monocyte chemoattractant protein-1
MMP: Matrix metalloproteinase
MS: Multiple sclerosis
NF-κB: Nuclear factor kappa-light-chain-enhancer of activated B cells
NK: Natural killer cell
NO: Nitric oxide
PBMC: Peripheral blood mononuclear cells
PK: Pharmacokinetics
PKA: Protein kinase A
PPAR: Peroxisome proliferator activated receptor
ROS: Reactive oxygen species
SARS-CoV-2: Severe acute respiratory syndrome-coronavirus-2
T1D: Type 1 diabetes
Th1: T-helper type 1
Th2: T-helper type 2
TLR: Toll-like receptor
T_reg_: Regulatory T cell
TRP: Transient receptor potential channel
Δ^9^-THC: Delta-9-tetrahydrocannabinol
Δ^9^-THCA: Delta-9-tetrahydrocannabinolic acid
VEA: Vitamin E acetate

## References

[1] Small E (1975). American law and the species problem in cannabis: science and semantics. Bull Narc.

[2] Merlin MD (2003). Archaeological evidence for the tradition of psychoactive plant use in the old world. Econ Bot.

[3] Baron EP (2018). Medicinal properties of cannabinoids, terpenes, and flavonoids in cannabis, and benefits in migraine, headache, and pain: an update on current evidence and cannabis science. Headache.

[4] Li H, Liu Y, Tian D, Tian L, Ju X, Qi L, Wang Y, Liang C (2020). Overview of cannabidiol (CBD) and its analogues: structures, biological activities, and neuroprotective mechanisms in epilepsy and Alzheimer’s disease. Eur J Med Chem.

[5] Brown JD (2020). Potential adverse drug events with tetrahydrocannabinol (THC) due to drug–drug interactions. J Clin Med.

[6] Campeny E, Lopez-Pelayo H, Nutt D, Blithikioti C, Oliveras C, Nuno L, Maldonado R, Florez G, Arias F, Fernandez-Artamendi S (2020). The blind men and the elephant: systematic review of systematic reviews of cannabis use related health harms. Eur Neuropsychopharmacol.

[7] Joshi M, Joshi A, Bartter T (2014). Marijuana and lung diseases. Curr Opin Pulm Med.

[8] Schuermeyer J, Salomonsen-Sautel S, Price RK, Balan S, Thurstone C, Min SJ, Sakai JT (2014). Temporal trends in marijuana attitudes, availability and use in Colorado compared to non-medical marijuana states: 2003–2011. Drug Alcohol Depend.

[9] Lake S, Kerr T, Werb D, Haines-Saah R, Fischer B, Thomas G, Walsh Z, Ware MA, Wood E, Milloy MJ (2019). Guidelines for public health and safety metrics to evaluate the potential harms and benefits of cannabis regulation in Canada. Drug Alcohol Rev.

[10] Welling MT, Shapter T, Rose TJ, Liu L, Stanger R, King GJ (2016). A belated green revolution for cannabis: virtual genetic resources to fast-track cultivar development. Front Plant Sci.

[11] Li H-L (1974). An archaeological and historical account of cannabis in China. Econ Bot.

[12] Touw M (1981). The religious and medicinal uses of cannabis in China, India and Tibet. J Psychoact Drugs.

[13] Li H-L (1978). Hallucinogenic plants in Chinese herbals. J Psychedelic Drugs.

[14] Mikuriya TH (1969). Marijuana in medicine: past, present and future. Calif Med.

[15] Mathre ML (1997). Cannabis in medical practice: a legal, historical and pharmacological overview of the therapeutic use of marijuana.

[16] Jiang H, Wang L, Merlin MD, Clarke RC, Pan Y, Zhang Y, Xiao G, Ding X (2016). Ancient cannabis burial shroud in a Central Eurasian Cemetery. Econ Bot.

[17] Du Toit BMUoFCfAS. Cannabis in Africa: a survey of its distribution in Africa, and a study of cannabis use and users in multi-et[h]nic South Africa. Rotterdam: Published for the African Studies Center, University of Florida, Gainesville, Florida by A.A. Balkema; 1980.

[18] O'Shaughnessy WB (1843). On the preparations of the Indian hemp, or Gunjah: *Cannabis indica* their effects on the animal system in health, and their utility in the treatment of tetanus and other convulsive diseases. Prov Med J Retrospect Med Sci.

[19] O'Shaughnessy WB (1844). The Bengal dispensatory and pharmacopoeia.

[20] O'Shaughnessy WB. The Bengal pharmacopoeia, and general conspectus of medicinal plants: arranged according to the natural and therapeutical systems: published by Order of Government. Bishop; 1844.

[21] Grinspoon L, Risani M, Fedeli R. Marihuana reconsidered. Apogeo; 1996.

[22] Wood TB, Spivey WTN, Easterfield TH (1899). III.—Cannabinol. Part I. J Chem Soc Trans.

[23] ElSohly MA, Radwan MM, Gul W, Chandra S, Galal A, Kinghorn AD, Falk H, Gibbons S, Kobayashi J (2017). Phytochemistry of *Cannabis sativa* L.. Phytocannabinoids: unraveling the complex chemistry and pharmacology of *Cannabis sativa*.

[24] Turner CE, Elsohly MA, Boeren EG (1980). Constituents of *Cannabis sativa* L. XVII. A review of the natural constituents. J Nat Prod.

[25] Zhou F, Pichersky E (2020). More is better: the diversity of terpene metabolism in plants. Curr Opin Plant Biol.

[26] Happyana N, Agnolet S, Muntendam R, Van Dam A, Schneider B, Kayser O (2013). Analysis of cannabinoids in laser-microdissected trichomes of medicinal *Cannabis sativa* using LCMS and cryogenic NMR. Phytochemistry.

[27] Sakamoto K, Shimomura K, Komeda Y, Kamada H, Satoh S (1995). A male-associated DNA sequence in a dioecious plant, *Cannabis sativa* L.. Plant Cell Physiol.

[28] Sirikantaramas S, Taura F, Morimoto S, Shoyama Y (2007). Recent advances in *Cannabis sativa* research: biosynthetic studies and its potential in biotechnology. Curr Pharm Biotechnol.

[29] Flores-Sánchez IJ, Verpoorte R (2008). Secondary metabolism in cannabis. Phytochem Rev.

[30] Small E, Cronquist A (1976). A practical and natural taxonomy for cannabis. Taxon.

[31] Gaoni Y, Mechoulam R (1964). Isolation, structure, and partial synthesis of an active constituent of hashish. J Am Chem Soc.

[32] Kandel DB (1984). Marijuana users in young adulthood. Arch Gen Psychiatry.

[33] The Canadian cannabis survey 2021: methodological report, Canada. Health Canada ib ed. Ottawa: Health Canada; 2021.

[34] Ware MA (2013). Cannabis and the lung: no more smoking gun?. Ann Am Thorac Soc.

[35] Hoffman D, Brunnemann KD, Gori GB, Wynder EL, Runeckles VC (1975). On the carcinogenicity of marijuana smoke. Recent advances in phytochemistry.

[36] Romero-Sandoval EA, Fincham JE, Kolano AL, Sharpe BN, Alvarado-Vazquez PA (2018). Cannabis for chronic pain: challenges and considerations. Pharmacotherapy.

[37] Grotenhermen F (2003). Pharmacokinetics and pharmacodynamics of cannabinoids. Clin Pharmacokinet.

[38] Parikh N, Kramer WG, Khurana V, Cognata Smith C, Vetticaden S (2016). Bioavailability study of dronabinol oral solution versus dronabinol capsules in healthy volunteers. Clin Pharmacol.

[39] Newmeyer MN, Swortwood MJ, Barnes AJ, Abulseoud OA, Scheidweiler KB, Huestis MA (2016). Free and glucuronide whole blood cannabinoids’ pharmacokinetics after controlled smoked, vaporized, and oral cannabis administration in frequent and occasional cannabis users: identification of recent cannabis intake. Clin Chem.

[40] Huestis MA (2007). Human cannabinoid pharmacokinetics. Chem Biodivers.

[41] Lucas CJ, Galettis P, Schneider J (2018). The pharmacokinetics and the pharmacodynamics of cannabinoids. Br J Clin Pharmacol.

[42] Hunt CA, Jones RT (1980). Tolerance and disposition of tetrahydrocannabinol in man. J Pharmacol Exp Ther.

[43] Nasrin S, Watson CJW, Perez-Paramo YX, Lazarus P (2021). Cannabinoid metabolites as inhibitors of major hepatic CYP450 enzymes, with implications for cannabis-drug interactions. Drug Metab Dispos.

[44] Devinsky O, Cilio MR, Cross H, Fernandez-Ruiz J, French J, Hill C, Katz R, Di Marzo V, Jutras-Aswad D, Notcutt WG (2014). Cannabidiol: pharmacology and potential therapeutic role in epilepsy and other neuropsychiatric disorders. Epilepsia.

[45] Devane WA, Dysarz FA, Johnson MR, Melvin LS, Howlett AC (1988). Determination and characterization of a cannabinoid receptor in rat brain. Mol Pharmacol.

[46] Matsuda LA, Lolait SJ, Brownstein MJ, Young AC, Bonner TI (1990). Structure of a cannabinoid receptor and functional expression of the cloned cDNA. Nature.

[47] Munro S, Thomas KL, Abu-Shaar M (1993). Molecular characterization of a peripheral receptor for cannabinoids. Nature.

[48] Devane WA, Hanus L, Breuer A, Pertwee RG, Stevenson LA, Griffin G, Gibson D, Mandelbaum A, Etinger A, Mechoulam R (1992). Isolation and structure of a brain constituent that binds to the cannabinoid receptor. Science.

[49] Wu J (2019). Cannabis, cannabinoid receptors, and endocannabinoid system: yesterday, today, and tomorrow. Acta Pharmacol Sin.

[50] Pertwee RG (2010). Receptors and channels targeted by synthetic cannabinoid receptor agonists and antagonists. Curr Med Chem.

[51] Pertwee RG (2015). Endocannabinoids and their pharmacological actions. Handb Exp Pharmacol.

[52] Starowicz K, Finn DP (2017). Cannabinoids and pain: sites and mechanisms of action. Adv Pharmacol.

[53] Walker JM, Huang SM (2002). Endocannabinoids in pain modulation. Prostaglandins Leukot Essent Fatty Acids.

[54] Lu H-C, Mackie K (2016). An introduction to the endogenous cannabinoid system. Biol Psychiat.

[55] Kano M (2014). Control of synaptic function by endocannabinoid-mediated retrograde signaling. Proc Jpn Acad Ser B Phys Biol Sci.

[56] Kreitzer AC, Regehr WG (2001). Retrograde inhibition of presynaptic calcium influx by endogenous cannabinoids at excitatory synapses onto Purkinje cells. Neuron.

[57] Hashimotodani Y, Ohno-Shosaku T, Kano M (2007). Presynaptic monoacylglycerol lipase activity determines basal endocannabinoid tone and terminates retrograde endocannabinoid signaling in the hippocampus. J Neurosci.

[58] Gonsiorek W, Lunn C, Fan X, Narula S, Lundell D, Hipkin RW (2000). Endocannabinoid 2-arachidonyl glycerol is a full agonist through human type 2 cannabinoid receptor: antagonism by anandamide. Mol Pharmacol.

[59] Luk T, Jin W, Zvonok A, Lu D, Lin XZ, Chavkin C, Makriyannis A, Mackie K (2004). Identification of a potent and highly efficacious, yet slowly desensitizing CB1 cannabinoid receptor agonist. Br J Pharmacol.

[60] Reggio PH, Panu AM, Miles S (1993). Characterization of a region of steric interference at the cannabinoid receptor using the active analog approach. J Med Chem.

[61] Milligan AL, Szabo-Pardi TA, Burton MD (2020). Cannabinoid receptor type 1 and its role as an analgesic: an opioid alternative?. J Dual Diagn.

[62] Bie B, Wu J, Foss JF, Naguib M (2018). An overview of the cannabinoid type 2 receptor system and its therapeutic potential. Curr Opin Anaesthesiol.

[63] Mackie K (2005). Distribution of cannabinoid receptors in the central and peripheral nervous system. Handbook of experimental pharmacology.

[64] Calignano A, Katona I, Desarnaud F, Giuffrida A, La Rana G, Mackie K, Freund TF, Piomelli D (2000). Bidirectional control of airway responsiveness by endogenous cannabinoids. Nature.

[65] Turcotte C, Blanchet M-R, Laviolette M, Flamand N (2016). Impact of cannabis, cannabinoids, and endocannabinoids in the lungs. Front Pharmacol.

[66] Tschop J, Kasten KR, Nogueiras R, Goetzman HS, Cave CM, England LG, Dattilo J, Lentsch AB, Tschop MH, Caldwell CC (2009). The cannabinoid receptor 2 is critical for the host response to sepsis. J Immunol.

[67] Liu Z, Wang Y, Zhao H, Zheng Q, Xiao L, Zhao M (2014). CB2 receptor activation ameliorates the proinflammatory activity in acute lung injury induced by paraquat. Biomed Res Int.

[68] Shang VC, Kendall DA, Roberts RE (2016). Delta9-Tetrahydrocannabinol reverses TNFalpha-induced increase in airway epithelial cell permeability through CB2 receptors. Biochem Pharmacol.

[69] Irving A, Abdulrazzaq G, Chan SLF, Penman J, Harvey J, Alexander SPH (2017). Cannabinoid receptor-related orphan G protein-coupled receptors. Adv Pharmacol.

[70] Ryberg E, Larsson N, Sjögren S, Hjorth S, Hermansson NO, Leonova J, Elebring T, Nilsson K, Drmota T, Greasley PJ (2007). The orphan receptor GPR55 is a novel cannabinoid receptor. Br J Pharmacol.

[71] Oka S, Nakajima K, Yamashita A, Kishimoto S, Sugiura T (2007). Identification of GPR55 as a lysophosphatidylinositol receptor. Biochem Biophys Res Commun.

[72] Gantz I, Muraoka A, Yang YK, Samuelson LC, Zimmerman EM, Cook H, Yamada T (1997). Cloning and chromosomal localization of a gene (GPR18) encoding a novel seven transmembrane receptor highly expressed in spleen and testis. Genomics.

[73] McHugh D, Page J, Dunn E, Bradshaw HB (2012). Δ(9)-Tetrahydrocannabinol and N-arachidonyl glycine are full agonists at GPR18 receptors and induce migration in human endometrial HEC-1B cells. Br J Pharmacol.

[74] Hansen KB, Rosenkilde MM, Knop FK, Wellner N, Diep TA, Rehfeld JF, Andersen UB, Holst JJ, Hansen HS (2011). 2-Oleoyl glycerol is a GPR119 agonist and signals GLP-1 release in humans. J Clin Endocrinol Metab.

[75] Godlewski G, Offertaler L, Wagner JA, Kunos G (2009). Receptors for acylethanolamides-GPR55 and GPR119. Prostaglandins Other Lipid Mediat.

[76] Jacobson KA, Gao ZG (2006). Adenosine receptors as therapeutic targets. Nat Rev Drug Discov.

[77] Borea PA, Gessi S, Merighi S, Vincenzi F, Varani K (2018). Pharmacology of adenosine receptors: the state of the art. Physiol Rev.

[78] Castillo A, Tolón MR, Fernández-Ruiz J, Romero J, Martinez-Orgado J (2010). The neuroprotective effect of cannabidiol in an in vitro model of newborn hypoxic-ischemic brain damage in mice is mediated by CB(2) and adenosine receptors. Neurobiol Dis.

[79] Hegde VL, Nagarkatti PS, Nagarkatti M (2011). Role of myeloid-derived suppressor cells in amelioration of experimental autoimmune hepatitis following activation of TRPV1 receptors by cannabidiol. PLoS ONE.

[80] Iannotti FA, Hill CL, Leo A, Alhusaini A, Soubrane C, Mazzarella E, Russo E, Whalley BJ, Di Marzo V, Stephens GJ (2014). Nonpsychotropic plant cannabinoids, cannabidivarin (CBDV) and cannabidiol (CBD), activate and desensitize transient receptor potential vanilloid 1 (TRPV1) channels in vitro: potential for the treatment of neuronal hyperexcitability. ACS Chem Neurosci.

[81] O'Sullivan SE (2016). An update on PPAR activation by cannabinoids. Br J Pharmacol.

[82] Sun Y, Bennett A (2007). Cannabinoids: a new group of agonists of PPARs. PPAR Res.

[83] Malfait AM, Gallily R, Sumariwalla PF, Malik AS, Andreakos E, Mechoulam R, Feldmann M (2000). The nonpsychoactive cannabis constituent cannabidiol is an oral anti-arthritic therapeutic in murine collagen-induced arthritis. Proc Natl Acad Sci USA.

[84] Arévalo-Martín A, Vela JM, Molina-Holgado E, Borrell J, Guaza C (2003). Therapeutic action of cannabinoids in a murine model of multiple sclerosis. J Neurosci.

[85] Docagne F, Muñetón V, Clemente D, Ali C, Loría F, Correa F, Hernangómez M, Mestre L, Vivien D, Guaza C (2007). Excitotoxicity in a chronic model of multiple sclerosis: neuroprotective effects of cannabinoids through CB1 and CB2 receptor activation. Mol Cell Neurosci.

[86] Schicho R, Storr M (2012). Topical and systemic cannabidiol improves trinitrobenzene sulfonic acid colitis in mice. Pharmacology.

[87] Jamontt JM, Molleman A, Pertwee RG, Parsons ME (2010). The effects of Delta-tetrahydrocannabinol and cannabidiol alone and in combination on damage, inflammation and in vitro motility disturbances in rat colitis. Br J Pharmacol.

[88] Storr MA, Keenan CM, Zhang H, Patel KD, Makriyannis A, Sharkey KA (2009). Activation of the cannabinoid 2 receptor (CB2) protects against experimental colitis. Inflamm Bowel Dis.

[89] Singh UP, Singh NP, Singh B, Price RL, Nagarkatti M, Nagarkatti PS (2012). Cannabinoid receptor-2 (CB2) agonist ameliorates colitis in IL-10(−/−) mice by attenuating the activation of T cells and promoting their apoptosis. Toxicol Appl Pharmacol.

[90] Lehmann C, Fisher NB, Tugwell B, Szczesniak A, Kelly M, Zhou J (2016). Experimental cannabidiol treatment reduces early pancreatic inflammation in type 1 diabetes. Clin Hemorheol Microcirc.

[91] Weiss L, Zeira M, Reich S, Har-Noy M, Mechoulam R, Slavin S, Gallily R (2006). Cannabidiol lowers incidence of diabetes in non-obese diabetic mice. Autoimmunity.

[92] Weiss L, Zeira M, Reich S, Slavin S, Raz I, Mechoulam R, Gallily R (2008). Cannabidiol arrests onset of autoimmune diabetes in NOD mice. Neuropharmacology.

[93] Li X, Kaminski NE, Fischer LJ (2001). Examination of the immunosuppressive effect of delta9-tetrahydrocannabinol in streptozotocin-induced autoimmune diabetes. Int Immunopharmacol.

[94] Cooper MA, Colonna M, Yokoyama WM (2009). Hidden talents of natural killers: NK cells in innate and adaptive immunity. EMBO Rep.

[95] Srivastava MD, Srivastava BI, Brouhard B (1998). Delta9 tetrahydrocannabinol and cannabidiol alter cytokine production by human immune cells. Immunopharmacology.

[96] Patel V, Borysenko M, Kumar MSA, Millard WJ (1985). Effects of acute and subchronic Δ9-tetrahydrocannabinol administration on the plasma catecholamine, β-endorphin, and corticosterone levels and splenic natural killer cell activity in rats. Proc Soc Exp Biol Med.

[97] Klein TW, Newton C, Friedman H (1987). Inhibition of natural killer cell function by marijuana components. J Toxicol Environ Health.

[98] Specter SC, Klein TW, Newton C, Mondragon M, Widen R, Friedman H (1986). Marijuana effects on immunity: suppression of human natural killer cell activity of delta-9-tetrahydrocannabinol. Int J Immunopharmacol.

[99] Massi P, Fuzio D, Viganò D, Sacerdote P, Parolaro D (2000). Relative involvement of cannabinoid CB(1) and CB(2) receptors in the Delta(9)-tetrahydrocannabinol-induced inhibition of natural killer activity. Eur J Pharmacol.

[100] Galli SJ, Borregaard N, Wynn TA (2011). Phenotypic and functional plasticity of cells of innate immunity: macrophages, mast cells and neutrophils. Nat Immunol.

[101] Giacalone VD, Margaroli C, Mall MA, Tirouvanziam R (2020). Neutrophil adaptations upon recruitment to the lung: new concepts and implications for homeostasis and disease. Int J Mol Sci.

[102] Naccache PH, Volpi M, Becker EL, Makryannis A, Sha'afi RI (1982). Cannabinoid induced degranulation of rabbit neutrophils. Biochem Biophys Res Commun.

[103] Kraft B, Wintersberger W, Kress HG (2004). Cannabinoid receptor-independent suppression of the superoxide generation of human neutrophils (PMN) by CP55 940, but not by anandamide. Life Sci.

[104] Maccarrone M, Fiorucci L, Erba F, Bari M, Finazzi-Agrò A, Ascoli F (2000). Human mast cells take up and hydrolyze anandamide under the control of 5-lipoxygenase and do not express cannabinoid receptors. FEBS Lett.

[105] Samson MT, Small-Howard A, Shimoda LM, Koblan-Huberson M, Stokes AJ, Turner H (2003). Differential roles of CB1 and CB2 cannabinoid receptors in mast cells. J Immunol.

[106] Bueb JL, Lambert DM, Tschirhart EJ (2001). Receptor-independent effects of natural cannabinoids in rat peritoneal mast cells in vitro. Biochim Biophys Acta.

[107] Lau AH, Chow SS (2003). Effects of cannabinoid receptor agonists on immunologically induced histamine release from rat peritoneal mast cells. Eur J Pharmacol.

[108] Matias I, Pochard P, Orlando P, Salzet M, Pestel J, Di Marzo V (2002). Presence and regulation of the endocannabinoid system in human dendritic cells. Eur J Biochem.

[109] Do Y, McKallip RJ, Nagarkatti M, Nagarkatti PS (2004). Activation through cannabinoid receptors 1 and 2 on dendritic cells triggers NF-kappaB-dependent apoptosis: novel role for endogenous and exogenous cannabinoids in immunoregulation. J Immunol.

[110] Roth MD, Castaneda JT, Kiertscher SM (2015). Exposure to Δ9-tetrahydrocannabinol impairs the differentiation of human monocyte-derived dendritic cells and their capacity for T cell activation. J Neuroimmune Pharmacol.

[111] Epelman S, Lavine KJ, Randolph GJ (2014). Origin and functions of tissue macrophages. Immunity.

[112] Mantovani A, Biswas SK, Galdiero MR, Sica A, Locati M (2013). Macrophage plasticity and polarization in tissue repair and remodelling. J Pathol.

[113] Deng YM, Zhao C, Wu L, Qu Z, Wang XY (2022). Cannabinoid receptor-1 suppresses M2 macrophage polarization in colorectal cancer by downregulating EGFR. Cell Death Discov.

[114] Braun M, Khan ZT, Khan MB, Kumar M, Ward A, Achyut BR, Arbab AS, Hess DC, Hoda MN, Baban B (2018). Selective activation of cannabinoid receptor-2 reduces neuroinflammation after traumatic brain injury via alternative macrophage polarization. Brain Behav Immun.

[115] Jiang L, Chen Y, Huang X, Yuan A, Shao Q, Pu J, He B (2016). Selective activation of CB2 receptor improves efferocytosis in cultured macrophages. Life Sci.

[116] Coffey RG, Snella E, Johnson K, Pross S (1996). Inhibition of macrophage nitric oxide production by tetrahydrocannabinol in vivo and in vitro. Int J Immunopharmacol.

[117] Tang JL, Lancz G, Specter S, Bullock H (1992). Marijuana and immunity: tetrahydrocannabinol-mediated inhibition of growth and phagocytic activity of the murine macrophage cell line, P388D1. Int J Immunopharmacol.

[118] Muthumalage T, Rahman I (2019). Cannabidiol differentially regulates basal and LPS-induced inflammatory responses in macrophages, lung epithelial cells, and fibroblasts. Toxicol Appl Pharmacol.

[119] Croxford JL, Yamamura T (2005). Cannabinoids and the immune system: potential for the treatment of inflammatory diseases?. J Neuroimmunol.

[120] Derocq JM, Ségui M, Marchand J, Le Fur G, Casellas P (1995). Cannabinoids enhance human B-cell growth at low nanomolar concentrations. FEBS Lett.

[121] Chen Y, Buck J (2000). Cannabinoids protect cells from oxidative cell death: a receptor-independent mechanism. J Pharmacol Exp Ther.

[122] Zhu W, Friedman H, Klein TW (1998). Delta9-tetrahydrocannabinol induces apoptosis in macrophages and lymphocytes: involvement of Bcl-2 and caspase-1. J Pharmacol Exp Ther.

[123] McKallip RJ, Lombard C, Fisher M, Martin BR, Ryu S, Grant S, Nagarkatti PS, Nagarkatti M (2002). Targeting CB2 cannabinoid receptors as a novel therapy to treat malignant lymphoblastic disease. Blood.

[124] Lombard C, Nagarkatti M, Nagarkatti P (2007). CB2 cannabinoid receptor agonist, JWH-015, triggers apoptosis in immune cells: potential role for CB2-selective ligands as immunosuppressive agents. Clin Immunol.

[125] Lee CY, Wey SP, Liao MH, Hsu WL, Wu HY, Jan TR (2008). A comparative study on cannabidiol-induced apoptosis in murine thymocytes and EL-4 thymoma cells. Int Immunopharmacol.

[126] Annunziata P, Cioni C, Mugnaini C, Corelli F (2017). Potent immunomodulatory activity of a highly selective cannabinoid CB2 agonist on immune cells from healthy subjects and patients with multiple sclerosis. J Neuroimmunol.

[127] Jordà MA, Verbakel SE, Valk PJ, Vankan-Berkhoudt YV, Maccarrone M, Finazzi-Agrò A, Löwenberg B, Delwel R (2002). Hematopoietic cells expressing the peripheral cannabinoid receptor migrate in response to the endocannabinoid 2-arachidonoylglycerol. Blood.

[128] Graves BM, Johnson TJ, Nishida RT, Dias RP, Savareear B, Harynuk JJ, Kazemimanesh M, Olfert JS, Boies AM (2020). Comprehensive characterization of mainstream marijuana and tobacco smoke. Sci Rep.

[129] Agrawal A, Budney AJ, Lynskey MT (2012). The co-occurring use and misuse of cannabis and tobacco: a review. Addiction.

[130] Meier E, Hatsukami DK (2016). A review of the additive health risk of cannabis and tobacco co-use. Drug Alcohol Depend.

[131] Tashkin DP, Gliederer F, Rose J, Chang P, Hui KK, Yu JL, Wu TC (1991). Tar, CO and delta 9THC delivery from the 1st and 2nd halves of a marijuana cigarette. Pharmacol Biochem Behav.

[132] Wu TC, Tashkin DP, Djahed B, Rose JE (1988). Pulmonary hazards of smoking marijuana as compared with tobacco. N Engl J Med.

[133] Tashkin DP, Baldwin GC, Sarafian T, Dubinett S, Roth MD (2002). Respiratory and immunologic consequences of marijuana smoking. J Clin Pharmacol.

[134] Roth MD, Arora A, Barsky SH, Kleerup EC, Simmons M, Tashkin DP (1998). Airway inflammation in young marijuana and tobacco smokers. Am J Respir Crit Care Med.

[135] Barbers RG, Gong H, Tashkin DP, Oishi J, Wallace JM (1987). Differential examination of bronchoalveolar lavage cells in tobacco cigarette and marijuana smokers. Am Rev Respir Dis.

[136] Gangwar RS, Vinayachandran V, Rengasamy P, Chan R, Park B, Diamond-Zaluski R, Cara EA, Cha A, Das L, Asase C (2020). Differential contribution of bone marrow-derived infiltrating monocytes and resident macrophages to persistent lung inflammation in chronic air pollution exposure. Sci Rep.

[137] Schyns J, Bai Q, Ruscitti C, Radermecker C, De Schepper S, Chakarov S, Farnir F, Pirottin D, Ginhoux F, Boeckxstaens G (2019). Non-classical tissue monocytes and two functionally distinct populations of interstitial macrophages populate the mouse lung. Nat Commun.

[138] Kawano H, Kayama H, Nakama T, Hashimoto T, Umemoto E, Takeda K (2016). IL-10-producing lung interstitial macrophages prevent neutrophilic asthma. Int Immunol.

[139] Helyes Z, Kemény Á, Csekő K, Szőke É, Elekes K, Mester M, Sándor K, Perkecz A, Kereskai L, Márk L (2017). Marijuana smoke induces severe pulmonary hyperresponsiveness, inflammation, and emphysema in a predictive mouse model not via CB1 receptor activation. Am J Physiol Lung Cell Mol Physiol.

[140] Fantauzzi MF, Cass SP, McGrath JJC, Thayaparan D, Wang P, Stampfli MR, Hirota JA (2021). Development and validation of a mouse model of contemporary cannabis smoke exposure. ERJ Open Res.

[141] Fligiel SE, Venkat H, Gong H, Tashkin DP (1988). Bronchial pathology in chronic marijuana smokers: a light and electron microscopic study. J Psychoact Drugs.

[142] Baldwin GC, Tashkin DP, Buckley DM, Park AN, Dubinett SM, Roth MD (1997). Marijuana and cocaine impair alveolar macrophage function and cytokine production. Am J Respir Crit Care Med.

[143] Tashkin DP (2013). Effects of marijuana smoking on the lung. Ann Am Thorac Soc.

[144] Botturi K, Langelot M, Lair D, Pipet A, Pain M, Chesne J, Hassoun D, Lacoeuille Y, Cavailles A, Magnan A (2011). Preventing asthma exacerbations: what are the targets?. Pharmacol Ther.

[145] Tashkin DP, Shapiro BJ, Frank IM (1974). Acute effects of smoked marijuana and oral delta9-tetrahydrocannabinol on specific airway conductance in asthmatic subjects. Am Rev Respir Dis.

[146] Ocampo TL, Rans TS (2015). *Cannabis sativa*: the unconventional “weed” allergen. Ann Allergy Asthma Immunol.

[147] Joshi M, Joshi A, Bartter T (2022). Marijuana and the lung: evolving understandings. Med Clin North Am.

[148] Tan WC, Lo C, Jong A, Xing L, Fitzgerald MJ, Vollmer WM, Buist SA, Sin DD (2009). Marijuana and chronic obstructive lung disease: a population-based study. CMAJ.

[149] Aldington S, Williams M, Nowitz M, Weatherall M, Pritchard A, McNaughton A, Robinson G, Beasley R (2007). Effects of cannabis on pulmonary structure, function and symptoms. Thorax.

[150] Tashkin DP (2018). Marijuana and lung disease. Chest.

[151] Gracie K, Hancox RJ (2021). Cannabis use disorder and the lungs. Addiction.

[152] Henry JA, Oldfield WLG, Kon OM (2003). Comparing cannabis with tobacco. BMJ.

[153] McCubbrey AL, Curtis JL (2013). Efferocytosis and lung disease. Chest.

[154] Tajbakhsh A, Gheibihayat SM, Mortazavi D, Medhati P, Rostami B, Savardashtaki A, Momtazi-Borojeni AA (2021). The effect of cigarette smoke exposure on efferocytosis in chronic obstructive pulmonary disease; molecular mechanisms and treatment opportunities. COPD.

[155] Liu Y, Liu H, Li C, Ma C, Ge W (2020). Proteome profiling of lung tissues in chronic obstructive pulmonary disease (COPD): platelet and macrophage dysfunction contribute to the pathogenesis of COPD. Int J Chron Obstruct Pulm Dis.

[156] Fligiel SE, Beals TF, Tashkin DP, Paule MG, Scallet AC, Ali SF, Bailey JR, Slikker W (1991). Marijuana exposure and pulmonary alterations in primates. Pharmacol Biochem Behav.

[157] Zhang LR, Morgenstern H, Greenland S, Chang SC, Lazarus P, Teare MD, Woll PJ, Orlow I, Cox B (2015). Cannabis smoking and lung cancer risk: pooled analysis in the international lung cancer consortium. Int J Cancer.

[158] Sidney S, Quesenberry CP, Friedman GD, Tekawa IS (1997). Marijuana use and cancer incidence (California, United States). Cancer Causes Control.

[159] Kaplan AG (2021). Cannabis and lung health: does the bad outweigh the good?. Pulm Ther.

[160] Talaska G, Schamer M, Bailey JR, Ali SF, Scallet AC, Slikker W, Paule MG (1992). No increase in carcinogen-DNA adducts in the lungs of monkeys exposed chronically to marijuana smoke. Toxicol Lett.

[161] Chatkin JM, Zani-Silva L, Ferreira I, Zamel N (2019). Cannabis-associated asthma and allergies. Clin Rev Allergy Immunol.

[162] Urban T, Hureaux J (2017). Cannabis et poumon. Ce que l’on sait et tout ce que l’on ne sait pas. Rev Pneumol Clin.

[163] José RJ (2018). Respiratory infections: a global burden. Ann Res Hosp.

[164] Quan DH, Kwong AJ, Hansbro PM, Britton WJ (2022). No smoke without fire: the impact of cigarette smoking on the immune control of tuberculosis. Eur Respir Rev.

[165] Kark JD, Lebiush M, Rannon L (1982). Cigarette smoking as a risk factor for epidemic a(h1n1) influenza in young men. N Engl J Med.

[166] Finklea JF, Sandifer SH, Smith DD (1969). Cigarette smoking and epidemic influenza. Am J Epidemiol.

[167] Godoy P, Castilla J, Soldevila N, Mayoral JM, Toledo D, Martín V, Astray J, Egurrola M, Morales-Suarez-Varela M, Domínguez A (2018). Smoking may increase the risk of influenza hospitalization and reduce influenza vaccine effectiveness in the elderly. Eur J Public Health.

[168] Chavez J, Hai R (2021). Effects of cigarette smoking on influenza virus/host interplay. Pathogens.

[169] Maggirwar SB, Khalsa JH (2021). The link between cannabis use, immune system, and viral infections. Viruses.

[170] Binns C, Low WY, Kyung LM (2020). The COVID-19 pandemic: public health and epidemiology. Asia Pac J Public Health.

[171] Morganstein T, Haidar Z, Trivlidis J, Azuelos I, Huang MJ, Eidelman DH, Baglole CJ (2021). Involvement of the ACE2/Ang-(1–7)/MasR axis in pulmonary fibrosis: implications for COVID-19. Int J Mol Sci.

[172] Rauf A, Abu-Izneid T, Olatunde A, Ahmed Khalil A, Alhumaydhi FA, Tufail T, Shariati MA, Rebezov M, Almarhoon ZM, Mabkhot YN (2020). COVID-19 pandemic: epidemiology, etiology, conventional and non-conventional therapies. Int J Environ Res Public Health.

[173] Kai H, Kai M (2020). Interactions of coronaviruses with ACE2, angiotensin II, and RAS inhibitors-lessons from available evidence and insights into COVID-19. Hypertens Res.

[174] Benowitz NL, Goniewicz ML, Halpern-Felsher B, Krishnan-Sarin S, Ling PM, O'Connor RJ, Pentz MA, Robertson RM, Bhatnagar A (2022). Tobacco product use and the risks of SARS-CoV-2 infection and COVID-19: current understanding and recommendations for future research. Lancet Respir Med.

[175] Aloufi N, Traboulsi H, Ding J, Fonseca GJ, Nair P, Huang SK, Hussain SNA, Eidelman DH, Baglole CJ (2021). Angiotensin-converting enzyme 2 expression in COPD and IPF fibroblasts: the forgotten cell in COVID-19. Am J Physiol Lung Cell Mol Physiol.

[176] Liu A, Zhang X, Li R, Zheng M, Yang S, Dai L, Wu A, Hu C, Huang Y, Xie M, Chen Q (2021). Overexpression of the SARS-CoV-2 receptor ACE2 is induced by cigarette smoke in bronchial and alveolar epithelia. J Pathol.

[177] Wang B, Kovalchuk A, Li D, Rodriguez-Juarez R, Ilnytskyy Y, Kovalchuk I, Kovalchuk O (2020). In search of preventive strategies: novel high-CBD *Cannabis sativa* extracts modulate ACE2 expression in COVID-19 gateway tissues. Aging (Albany NY).

[178] Nguyen LC, Yang D, Nicolaescu V, Best TJ, Gula H, Saxena D, Gabbard JD, Chen SN, Ohtsuki T, Friesen JB (2022). Cannabidiol inhibits SARS-CoV-2 replication through induction of the host ER stress and innate immune responses. Sci Adv.

[179] Anil SM, Shalev N, Vinayaka AC, Nadarajan S, Namdar D, Belausov E, Shoval I, Mani KA, Mechrez G, Koltai H (2021). Cannabis compounds exhibit anti-inflammatory activity in vitro in COVID-19-related inflammation in lung epithelial cells and pro-inflammatory activity in macrophages. Sci Rep.

[180] Paland N, Pechkovsky A, Aswad M, Hamza H, Popov T, Shahar E, Louria-Hayon I (2021). The immunopathology of COVID-19 and the cannabis paradigm. Front Immunol.

[181] Johnson ER, Matthay MA (2010). Acute lung injury: epidemiology, pathogenesis, and treatment. J Aerosol Med Pulm Drug Deliv.

[182] Ribeiro A, Ferraz-de-Paula V, Pinheiro ML, Vitoretti LB, Mariano-Souza DP, Quinteiro-Filho WM, Akamine AT, Almeida VI, Quevedo J, Dal-Pizzol F (2012). Cannabidiol, a non-psychotropic plant-derived cannabinoid, decreases inflammation in a murine model of acute lung injury: role for the adenosine A2A receptor. Eur J Pharmacol.

[183] Rao R, Nagarkatti PS, Nagarkatti M (2015). Δ9Tetrahydrocannabinol attenuates Staphylococcal enterotoxin B-induced inflammatory lung injury and prevents mortality in mice by modulation of miR-17-92 cluster and induction of T-regulatory cells. Br J Pharmacol.

[184] Berdyshev E, Boichot E, Corbel M, Germain N, Lagente V (1998). Effects of cannabinoid receptor ligands on LPS-induced pulmonary inflammation in mice. Life Sci.

[185] Karmaus PWF, Wagner JG, Harkema JR, Kaminski NE, Kaplan BLF (2013). Cannabidiol (CBD) enhances lipopolysaccharide (LPS)-induced pulmonary inflammation in C57BL/6 mice. J Immunotoxicol.

[186] Khodadadi H, Salles ÉL, Jarrahi A, Chibane F, Costigliola V, Yu JC, Vaibhav K, Hess DC, Dhandapani KM, Baban B (2020). Cannabidiol modulates cytokine storm in acute respiratory distress syndrome induced by simulated viral infection using synthetic RNA. Cannabis Cannabinoid Res.

[187] Chadi N, Minato C, Stanwick R (2020). Cannabis vaping: understanding the health risks of a rapidly emerging trend. Paediatr Child Health.

[188] Palamar JJ (2021). Increases in frequent vaping of cannabis among high school seniors in the United States, 2018–2019. J Adolesc Health.

[189] Fischer B, Russell C, Sabioni P, van den Brink W, Le Foll B, Hall W, Rehm J, Room R (2017). Lower-risk cannabis use guidelines: a comprehensive update of evidence and recommendations. Am J Public Health.

[190] Ryter SW, Choi AM (2013). Carbon monoxide in exhaled breath testing and therapeutics. J Breath Res.

[191] Abrams DI, Vizoso HP, Shade SB, Jay C, Kelly ME, Benowitz NL (2007). Vaporization as a smokeless cannabis delivery system: a pilot study. Clin Pharmacol Ther.

[192] Van Dam NT, Earleywine M (2010). Pulmonary function in cannabis users: support for a clinical trial of the vaporizer. Int J Drug Policy.

[193] Conuel EJ, Chieng HC, Fantauzzi J, Pokhrel K, Goldman C, Smith TC, Tiwari A, Chopra A, Judson MA (2020). Cannabinoid oil vaping-associated lung injury and its radiographic appearance. Am J Med.

[194] Lung illnesses associated with use of vaping products. https://www.fda.gov/news-events/public-health-focus/lung-illnesses-associated-use-vaping-products#Analysis.

[195] Matsumoto S, Fang X, Traber MG, Jones KD, Langelier C, Hayakawa Serpa P, Calfee CS, Matthay MA, Gotts JE (2020). Dose-dependent pulmonary toxicity of aerosolized vitamin E acetate. Am J Respir Cell Mol Biol.

[196] Shapouri-Moghaddam A, Mohammadian S, Vazini H, Taghadosi M, Esmaeili SA, Mardani F, Seifi B, Mohammadi A, Afshari JT, Sahebkar A (2018). Macrophage plasticity, polarization, and function in health and disease. J Cell Physiol.

